# Periosteal stem cells control growth plate stem cells during postnatal skeletal growth

**DOI:** 10.1038/s41467-022-31592-x

**Published:** 2022-07-18

**Authors:** Masayuki Tsukasaki, Noriko Komatsu, Takako Negishi-Koga, Nam Cong-Nhat Huynh, Ryunosuke Muro, Yutaro Ando, Yuka Seki, Asuka Terashima, Warunee Pluemsakunthai, Takeshi Nitta, Takashi Nakamura, Tomoki Nakashima, Shinsuke Ohba, Haruhiko Akiyama, Kazuo Okamoto, Roland Baron, Hiroshi Takayanagi

**Affiliations:** 1 Department of Immunology, Graduate School of Medicine and Faculty of Medicine, The University of Tokyo, 7-3-1, Hongo, Bunkyo-ku, 113-0033 Tokyo Japan; 2 Department of Community Medicine and Research for Bone and Joint Diseases, Juntendo University Graduate School of Medicine, 2-1-1, Hongo, Bunkyo-ku, 113-8421 Tokyo Japan; 3 Laboratory of Oral-Maxillofacial Biology, Faculty of Odonto-Stomatology, University of Medicine and Pharmacy at Ho Chi Minh City, Ho Chi Minh City, 749000 Viet Nam; 4 Department of Microbiology, Tokyo Dental College, 2-9-18, Kanda-Misakicho, Chiyoda-ku, 101-0061 Tokyo Japan; 5 Department of Osteoimmunology, Graduate School of Medicine and Faculty of Medicine, The University of Tokyo, 7-3-1, Hongo, Bunkyo-ku, 113-0033 Tokyo Japan; 6 Bone and Cartilage Regenerative Medicine, The University of Tokyo Hospital, 7-3-1, Hongo, Bunkyo-ku, 113-0033 Tokyo Japan; 7 Department of Biochemistry, Tokyo Dental College, 2-9-18, Kanda-Misakicho, Chiyoda-ku, 101-0061 Tokyo Japan; 8 Department of Cell Signaling, Graduate School of Medical and Dental Sciences, Tokyo Medical and Dental University, 1-5-45, Yushima, Bunkyo-ku, 113-8549 Tokyo Japan; 9 Department of Cell Biology, Institute of Biomedical Sciences, Nagasaki University, 1-7-1 Sakamoto, 852-8588 Nagasaki, Japan; 10 Department of Orthopaedic Surgery, School of Medicine, Gifu University, 1-1 Yanagido, 501-1194 Gifu City, Japan; 11 Division of Bone and Mineral Research, Oral Medicine, Infection and Immunity, Harvard School of Dental Medicine, Boston, MA USA; 12 Department of Medicine, Harvard Medical School and Endocrine Unit, MGH, Boston, MA USA; 13 Present Address: Department of Oral Anatomy and Developmental Biology, Graduate School of Dentistry, Osaka University, 1-8 Yamadaoka, Suita, Osaka 565-0871 Japan

**Keywords:** Bone, Bone development, Osteoimmunology, Mesenchymal stem cells

## Abstract

The ontogeny and fate of stem cells have been extensively investigated by lineage-tracing approaches. At distinct anatomical sites, bone tissue harbors multiple types of skeletal stem cells, which may independently supply osteogenic cells in a site-specific manner. Periosteal stem cells (PSCs) and growth plate resting zone stem cells (RZSCs) critically contribute to intramembranous and endochondral bone formation, respectively. However, it remains unclear whether there is functional crosstalk between these two types of skeletal stem cells. Here we show PSCs are not only required for intramembranous bone formation, but also for the growth plate maintenance and prolonged longitudinal bone growth. Mice deficient in PSCs display progressive defects in intramembranous and endochondral bone formation, the latter of which is caused by a deficiency in PSC-derived Indian hedgehog (Ihh). PSC-specific deletion of Ihh impairs the maintenance of the RZSCs, leading to a severe defect in endochondral bone formation in postnatal life. Thus, crosstalk between periosteal and growth plate stem cells is essential for post-developmental skeletal growth.

## Abstract (web summary)

Intramembranous and endochondral bone formation have been considered to be independent processes mediated by independent stem cells. Here the authors show that periosteal stem cells participate in both types of bone formation, supporting endochondral formation by producing Ihh.

## Introduction

The skeleton houses at least three sources of skeletal stem cells: the resting zone of the growth plate, the bone marrow and the periosteum/perichondrium^1–5^. Each of these stem cells has specific characteristics and contributes to bone homeostasis by serving as the local source of osteogenic cells and recent studies suggest an ontogenic hierarchy among these skeletal stem cells^1,2,5^. Mammalian bone is formed through two distinct growth processes: endochondral and intramembranous bone formation^6^. In endochondral bone formation, parathyroid hormone-related protein (PTHrP)-expressing RZSCs are the source of growth plate chondrocytes, which give rise to bone-forming osteoblasts as well as bone marrow skeletal stem cells^1^. Growth plate chondrocytes undergo differentiation into hypertrophic chondrocytes, which express Ihh, the master regulator of endochondral bone formation that controls chondrocyte proliferation, maturation and osteoblast differentiation^1,2,5,6^. Although conditional deletion of *Ihh* by means of *Col2a1*-Cre leads to a severe defect in bone development^7^, the fact that most of the mesenchymal lineage cells in bone originate from *Col2a1*-expressing cells^8^, raises the possibility that the cellular source of Ihh may not be limited to hypertrophic chondrocytes and could include the periosteum for instance.

A recent study identified cathepsin K (*Ctsk*)-Cre expressing PSCs as a unique class of skeletal stem cells residing in the periosteum^3^. PSCs are considered to exert their effects exclusively in intramembranous bone formation, since *Ctsk*-Cre-mediated deletion of *Sp7*, an essential transcriptional factor for osteoblast differentiation, impacted intramembranous but not endochondral bone formation^3^. In this system however, osteoblast differentiation of PSCs is selectively inhibited by *Sp7* deficiency but PSCs are still present and may still play a role in skeletal growth.

Here we show that PSCs not only regulate intramembranous bone formation but also contribute significantly to endochondral bone formation. When exploring the role of protein arginine methyltransferase 5 (PRMT5) in osteoclasts, we serendipitously developed a mouse model in which PSCs are specifically abrogated. These mice display progressive defects in intramembranous and endochondral bone formation, the latter of which is caused by a deficiency in PSC-derived Ihh. Thus, the periosteum/perichondrium and the growth plate are engaged in a specific Ihh-dependent crosstalk by which the growth plate-derived Ihh acts on the periosteum/perichondrium at early stages of development^9–11^ and, as shown here, the periosteum/perichondrium-derived Ihh maintains growth plate homeostasis and ensures the skeletal growth in postnatal stages.

## Results

### *Ctsk*-Cre-mediated deletion of *Prmt5* impacts bone growth

**Fig. 1:**
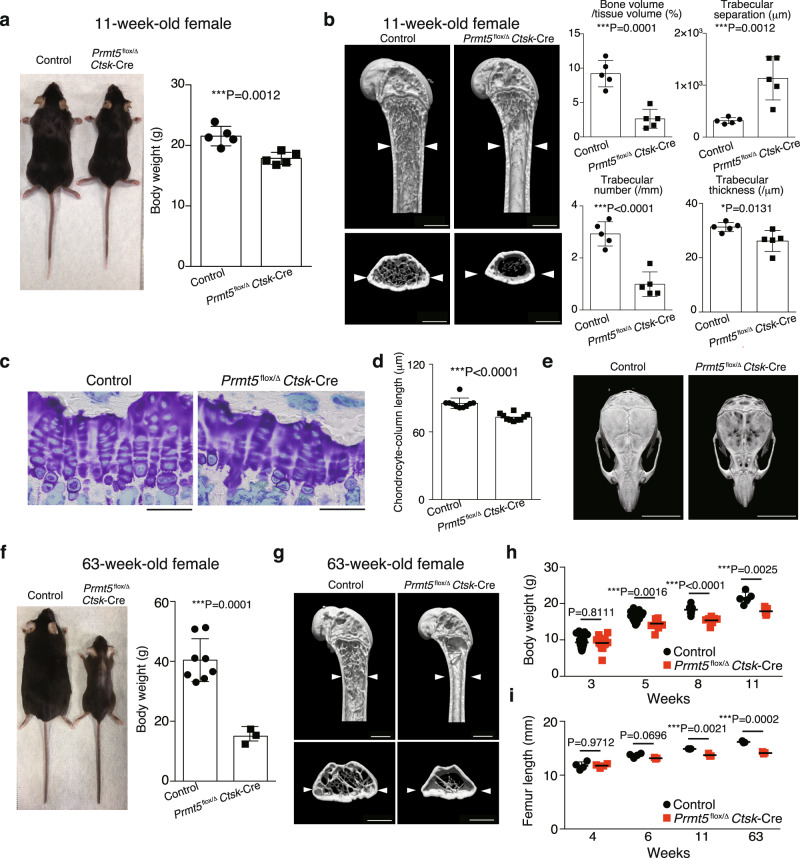
Impaired bone growth in *Prmt5*^flox/Δ^*Ctsk*-Cre mice. **a** Macroscopic image and body weight of *Prmt5*^flox/+^ *Ctsk*-Cre and *Prmt5*^flox/Δ^ *Ctsk*-Cre mice (*n* = 5 mice per group). *P* value was calculated using one-sided Student’s *t*-test. Data are presented as the mean ± S.D. **b** Representative μCT images and parameters of the femur in *Prmt5*^flox/+^ *Ctsk*-Cre and *Prmt5*^flox/Δ^ *Ctsk*-Cre mice (n = 5 mice per group). The white arrow heads indicate bone width in the control mice. Scale bars, 1 mm. *P* value was calculated using one-sided Student’s *t*-test. Data are presented as the mean ± S.D. **c** Toluidine blue staining of the growth plates in the proximal tibiae of 11-week-old female *Prmt5*^flox/+^ *Ctsk*-Cre and *Prmt5*^flox/Δ^ *Ctsk*-Cre mice. Representative pictures of more than three independent experiments are shown. Scale bars, 50 μm. **d** Chondrocyte-column length in the growth plates in the proximal tibiae of 11-week-old female *Prmt5*^flox/+^ *Ctsk*-Cre and *Prmt5*^flox/Δ^ *Ctsk*-Cre mice (*n* = 10 mice per group). *P* value was calculated using one-sided Student’s *t*-test. Data are presented as the mean ± S.D. **e** Representative μCT images of the skull of more than three littermates at the age of 11 weeks. Scale bars, 7 mm. **f** Macroscopic image and body weight of female control and *Prmt5*^flox/Δ^ *Ctsk*-Cre (*n* = 8 and 3 mice per group) mice at 63 weeks of age. *Prmt5*^flox/+^, *Prmt5*^flox/Δ^, and *Prmt5*^flox/+^ *Ctsk*-Cre mice were phenotypically identical, and so were grouped together and used as controls in (**f**–**i**). *P* value was calculated using one-sided Student’s *t*-test. Data are presented as the mean ± S.D. **g** Representative μCT images of the femur of male littermates at 63 weeks of age. Representative pictures of more than three independent experiments are shown. The white arrow heads indicate bone width in the control mice. Scale bars, 1 mm. **h** Body weight of female littermates at 3, 5, 8 and 11 weeks of age (3 weeks: *n* = 21 and 13, 5 weeks: *n* = 16 and 9, 8 weeks: *n* = 10 and 7, 11 weeks: *n* = 5 mice per group). *P* values were calculated using one-sided Student’s *t*-test. Data are presented as the mean ± S.D. **i** Femur length in female littermates at 4, 6, 11 and 63 weeks of age (4 weeks: *n* = 4, 6 weeks: *n* = 4 and 3, 11 weeks: *n* = 3, 63 weeks: *n* = 3 mice per group). *P* values were calculated using one-sided Student’s *t*-test. Data are presented as the mean ± S.D. Source data are provided as a Source Data file.

PRMT5 is a major enzyme responsible for symmetrical demethylation of arginine on target proteins^12^. Previous studies showed that Prmt5 is essential for homeostasis of various types of stem cells, such as ES cells, neural stem/progenitor cells, muscle stem cells and hematopoietic stem cells, suggesting that the requirement of Prmt5 may be one of the common characteristics shared by stem cells^13^. To explore the role of PRMT5 in bone homeostasis, and because our initial interest was osteoclastogenesis, we crossed *Prmt5*^flox/Δ^ mice^12^ with *Ctsk*-Cre mice^14^ in which Cre recombinase is expressed not only in osteoclasts but also in PSCs^3,15,16^. *Prmt5*^flox/Δ^ *Ctsk*-Cre mice were smaller than control mice in body size and exhibited a decrease in bone mass in the femur at the age of 11 weeks (Fig. 1a, b and Supplementary Fig. 1a, b). *Prmt5*^flox/Δ^ *Ctsk*-Cre mice displayed an abnormal growth plate architecture with a reduced chondrocyte column length, indicating impaired endochondral bone formation (Fig. 1c, d). These mice also exhibited a reduction in bone width and calvarial bone volume, suggesting that intramembranous bone formation was also affected in *Prmt5*^flox/Δ^ *Ctsk*-Cre mice (Fig. 1b, e). The altered skeletal phenotypes became more obvious with aging (Fig. 1f–i). Thus, *Prmt5*^flox/Δ^ *Ctsk*-Cre mice postnatally displayed a severe defect in endochondral and intramembranous bone formation. The number of hematopoietic stem and progenitor cells in the bone marrow dramatically decreased in *Prmt5*^flox/Δ^ *Ctsk*-Cre mice in the age of 40-63 weeks (Supplementary Fig. 1c). The serum concentrations of calcium and phosphate as well as serum parameters of nutritional status were normal in *Prmt5*^flox/Δ^ *Ctsk*-Cre mice (Supplementary Fig. 2).

### Osteoclast phenotypes in *Prmt5*^flox/Δ^*Ctsk*-Cre mice

Bone histomorphometric analyses and in vitro culture experiments showed that osteoclast differentiation and function were normal in *Prmt5*^flox/Δ^ *Ctsk*-Cre mice (Supplementary Fig. 3). RNA-seq analysis revealed that Prmt5-deficiency did not affect the gene expression profile in osteoclasts (Supplementary Fig. 3h, i). The *Prmt5* mRNA expression levels markedly decreased during osteoclastogenesis in bone marrow cells derived from *Prmt5*^flox/Δ^ *Ctsk*-Cre mice, whereas the expression levels of osteoclast marker genes (*Nfatc1*, *Acp5*, *Tnfrsf11a*, *Mmp9* and *Dcstamp*) did not change (Supplementary Fig. 3j). Furthermore, bone marrow transfer experiments revealed that osteoclasts were not responsible for the skeletal phenotypes in *Prmt5*^flox/Δ^ *Ctsk*-Cre mice (Supplementary Fig. 4a–j). To further exclude the possibility of osteoclast contribution to the Prmt5-deficiency phenotype, we crossed *Prmt5*^flox/flox^ mice with *LysM*-Cre mice^17^, in which Cre recombinase is expressed in myeloid cells including osteoclast precursors. *Prmt5*^flox/flox^ *LysM*-Cre mice displayed normal body size and no decrease in bone mass, bone length or bone width (Supplementary Fig. 4k–p). In contrast to *Prmt5*^flox/Δ^ *Ctsk*-Cre mice, *Prmt5*^flox/flox^ *LysM*-Cre mice showed a slightly increased bone volume, suggesting that Prmt5 may be required for homeostasis of early stage osteoclast precursors (Supplementary Fig. 4n). Thus, the low bone mass phenotype in *Prmt5*^flox/Δ^ *Ctsk*-Cre mice cannot be attributed to the Prmt5 deletion in osteoclast-lineage cells. We also crossed *Prmt5*^flox/flox^ mice with *Col2a1-*Cre mice^18^ and *Sp7*-Cre mice^19^, in both of which Cre recombinase is expressed in most of mesenchymal lineage cells in bone including the periosteum^8^. *Prmt5*^flox/flox^ *Col2a1-*Cre and *Prmt5*^flox/flox^ *Sp7*-Cre mice were smaller than control mice in body size and exhibited a decrease in bone mass, bone length and bone width, recapitulating the phenotypes of *Prmt5*^flox/Δ^ *Ctsk*-Cre mice (Supplementary Fig. 5). Collectively, these data indicated that Ctsk^+^ mesenchymal cells, but not osteoclasts, were responsible for the impaired bone growth in *Prmt5*^flox/Δ^ *Ctsk*-Cre mice.

### PSCs are selectively abrogated by Prmt5 deficiency

**Fig. 2:**
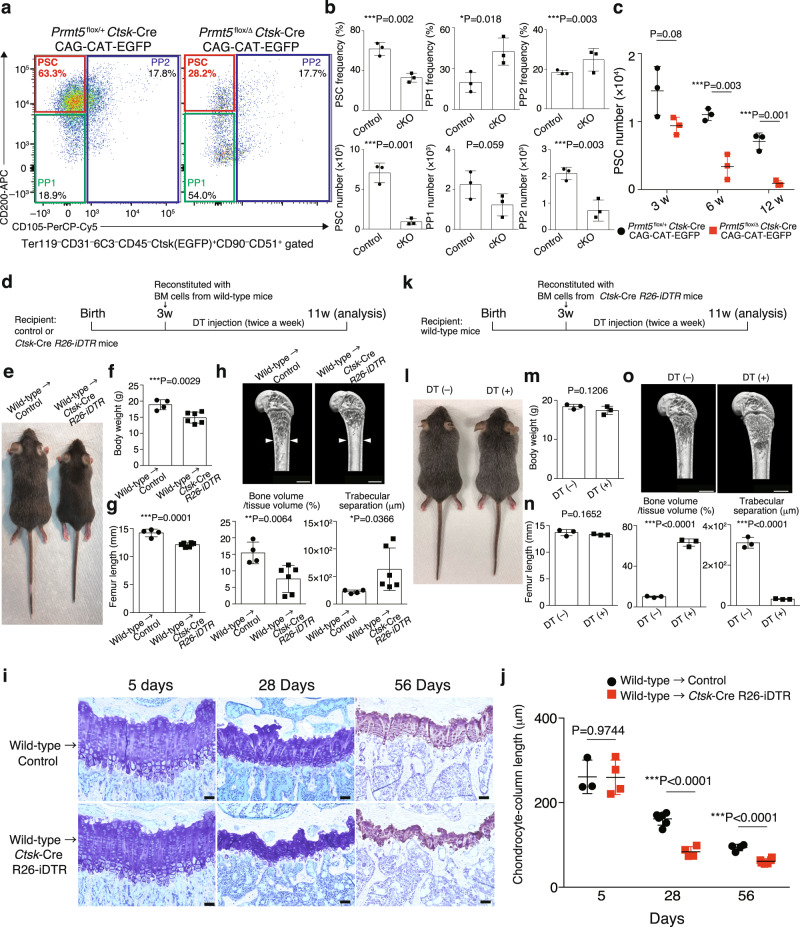
PSCs are abrogated by Prmt5-deficiency. **a** Representative FACS plots of analyses in (**b**). **b** The frequency and number of periosteal osteogenic progenitors in 12-week-old littermates (*n* = 3 mice per group). *P* values were calculated using one-sided Student’s *t*-test. Data are presented as the mean ± S.D. **c** PSC number in the littermates at multiple time points (*n* = 3 mice per group). *P* values were calculated using one-sided Student’s *t*-test. Data are presented as the mean ± S.D. **d** Experimental settings for (**e**–**h**) (**e**) Representative pictures of more than three bone marrow chimeric mice. **f** Body weight of bone marrow chimeric mice (*n* = 4 and 6 mice per group). *P* value was calculated using one-sided Student’s *t*-test. Data are presented as the mean ± S.D. **g** Femur length in bone marrow chimeric mice (*n* = 4 and 6 mice per group). *P* value was calculated using one-sided Student’s *t*-test. Data are presented as the mean ± S.D. **h** Representative μCT images and parameters of the femur in bone marrow chimeric mice (n = 4 and 6 mice per group). The white arrow heads indicate bone width in the control mice. Scale bars, 1 mm. *P* values were calculated using one-sided Student’s *t*-test. Data are presented as the mean ± S.D. **i** Representative pictures of the growth plates in control and *Ctsk*-Cre *R26*-*iDTR* mice at multiple time points after the bone marrow transfer and the first diphtheria toxin treatment. Representative images of more than three independent experiments are shown. Scale bars, 50 μm. **j** The length of chondrocyte-column presented in (**i**) was quantified (5 days: *n* = 3 and 4, 28 days: *n* = 6 and 4, 56 days: *n* = 4 and 6 mice per group). *P* values were calculated using one-sided Student’s *t*-test. Data are presented as the mean ± S.D. **k** Experimental settings for (**l**–**n**). **l** Representative pictures of more than three bone marrow chimeric mice treated with diphtheria toxin (DT+) or saline (DT−). **m** Body weight of bone marrow chimeric mice (*n* = 3 mice per group). *P* value was calculated using one-sided Student’s *t*-test. **n** Femur length in bone marrow chimeric mice (*n* = 3 mice per group). *P* value was calculated using one-sided Student’s *t*-test. **o** Representative μCT images and parameters of the femur in bone marrow chimeric mice (*n* = 3 mice per group). Scale bars, 1 mm. *P* values were calculated using one-sided Student’s *t*-test. Source data are provided as a Source Data file.

In *Ctsk*-Cre mice, Cre recombinase is reportedly expressed in osteogenic progenitors at the periosteum^3,15,16^. To detect periosteal osteogenic progenitors with EGFP, we crossed *Prmt5*^flox/Δ^ *Ctsk*-Cre mice or *Prmt5*^flox/+^ *Ctsk*-Cre mice with CAG-CAT-EGFP mice (Supplementary Fig. 6). A previous report showed that Ter119^–^CD31^−^6C3^−^CD45^−^CD90^−^Ctsk^+^CD51^+^ periosteal osteogenic progenitors are composed of CD200^+^CD105^−^ PSC and its descendants: CD200^–^CD105^–^ periosteal progenitor 1 (PP1) and CD200^variable^CD105^+^ periosteal progenitor 2 (PP2)^3^. The expression levels of Prmt5 mRNA and protein decreased in PSCs, but not in growth plate chondrocytes in *Prmt5*^flox/Δ^ *Ctsk*-Cre CAG-CAT-EGFP mice (Supplementary Fig. 7a, b). We found that the frequency and number of PSCs, but not PP1 or PP2, markedly decreased in *Prmt5*^flox/Δ^ *Ctsk*-Cre CAG-CAT-EGFP mice (Fig. 2a, b). Intriguingly, the number of PSCs progressively decreased with aging in *Prmt5*^flox/Δ^ *Ctsk*-Cre CAG-CAT-EGFP mice, consistent with an age-dependent skeletal phenotype (Fig. 2c).

### Ablation of PSCs impacts on endochondral bone formation

To confirm the effect of PSC deletion on bone growth, we crossed *Ctsk*-Cre mice with mice containing a Cre-inducible diphtheria toxin (DT) receptor (*Ctsk*-Cre *R26*-*iDTR* mice), allowing for the DT-mediated depletion of Ctsk^+^ cells. When we transferred wild-type bone marrow cells into *Ctsk*-Cre *R26*-*iDTR* mice at the age of 3 weeks and then treated the mice with DT for 8 weeks, these mice displayed reduced body size, body weight, bone length, bone width and trabecular bone volume (Fig. 2d–h). In *Ctsk*-Cre *R26*-*iDTR* CAG-CAT-EGFP mice transferred with wild-type bone marrow cells, PSCs were efficiently deleted 5 days after DT treatment, while other cell types including osteoclasts, osteoblasts and chondrocytes were not affected at this stage (Fig. 2i, j and Supplementary Fig. 7c–f). After four weeks of the PSC deletion, the mice exhibited an impaired periosteal bone formation with a compensatory increase in endosteal bone formation (Supplementary Fig. 7g, h), which is consistent with other mouse models having impaired intramembranous bone formation such as *Prmt5*^flox/Δ^ *Ctsk*-Cre mice (Supplementary Fig. 3c) and *Sp7*^flox/flox^ *Ctsk*-Cre mice^3^. Histological analyses showed that intramembranous (Supplementary Fig. 7h) and endochondral bone formation (Fig. 2i, j) were progressively impaired after the postnatal deletion of PSCs.

In contrast, this protocol resulted in a severe high bone mass phenotype due to osteoclast deficiency without any evident change in body size, body weight or bone length in wild-type mice transferred with *Ctsk*-Cre *R26*-*iDTR* bone marrow cells (Fig. 2k–o). Collectively, these findings suggest that PSCs play a key role in both endochondral- and intramembranous-dependent postnatal bone growth.

### Abnormal splicing of *Periostin* mRNA in Prmt5-deficient PSCs

**Fig. 3:**
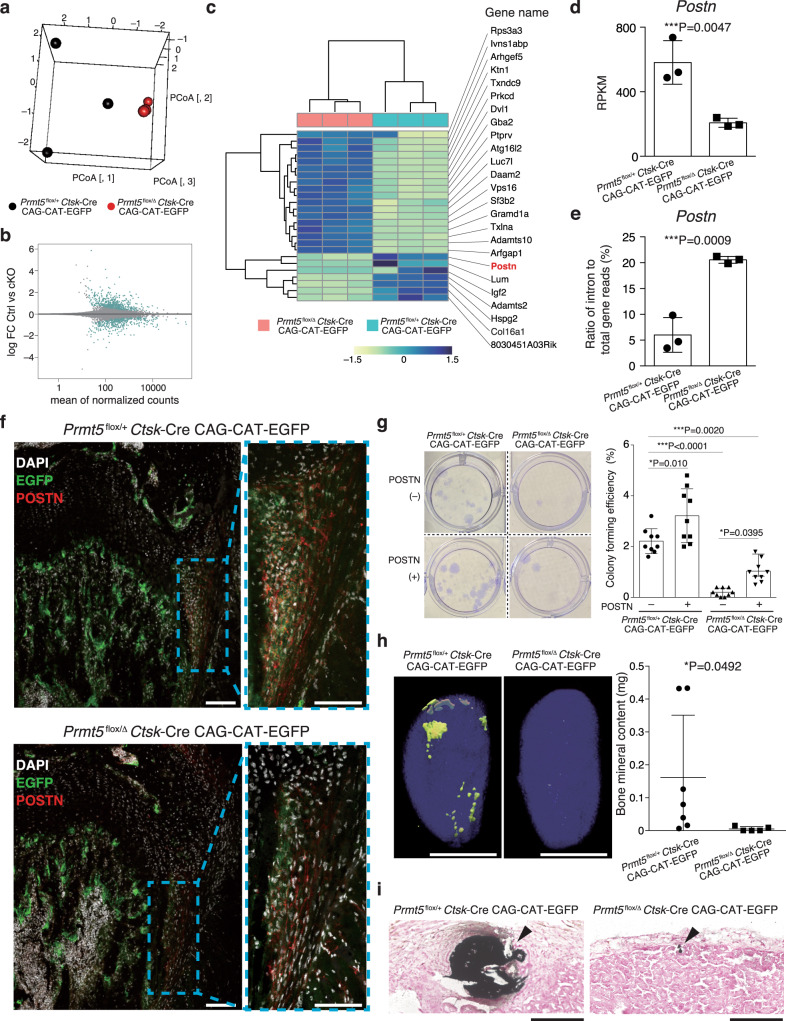
Abnormal *Periostin* mRNA splicing in Prmt5-deficient PSCs. **a** Principal coordinate analysis (PCoA) performed on PSCs derived from *Prmt5*^flox/Δ^ *Ctsk*-Cre CAG-CAT-EGFP and *Prmt5*^flox/+^ *Ctsk*-Cre CAG-CAT-EGFP mice (*n* = 3 mice per group). **b** MA plot of significant genes that were differentially expressed in the PSCs derived from *Prmt5*^flox/Δ^ *Ctsk*-Cre CAG-CAT-EGFP and *Prmt5*^flox/+^ *Ctsk*-Cre CAG-CAT-EGFP mice (light blue dots). **c** Heatmap of the top 25 genes of which expression was highly altered in PSCs by the *Prmt5*-deficiency. The color bar indicates scaled normalized gene expression counts. **d** The expression level of *Postn* mRNA in PSCs derived from *Prmt5*^flox/Δ^ *Ctsk*-Cre CAG-CAT-EGFP and *Prmt5*^flox/+^ *Ctsk*-Cre CAG-CAT-EGFP mice (*n* = 3 mice per group). *P* value was calculated using one-sided Student’s *t*-test. Data are presented as the mean ± S.D. **e** The ratio of intron reads to total gene reads in *Postn* gene in PSCs derived from *Prmt5*^flox/Δ^ *Ctsk*-Cre CAG-CAT-EGFP and *Prmt5*^flox/+^ *Ctsk*-Cre CAG-CAT-EGFP mice (*n* = 3 mice per group). *P* value was calculated using one-sided Student’s *t*-test. Data are presented as the mean ± S.D. **f** Immunohistochemical analysis of the femur of *Prmt5*^flox/Δ^ *Ctsk*-Cre CAG-CAT-EGFP and *Prmt5*^flox/+^ *Ctsk*-Cre CAG-CAT-EGFP mice. Representative images of more than three independent experiments are shown. Scale bars, 100 μm. **g** Colony formation of PSCs derived from *Prmt5*^flox/Δ^ *Ctsk*-Cre CAG-CAT-EGFP and *Prmt5*^flox/+^ *Ctsk*-Cre CAG-CAT-EGFP mice in the presence or absence of recombinant periostin (POSTN). Representative data of more than three independent experiments (left) and colony forming efficiency (right) are shown (*n* = 9 biologically independent samples). *P* values were calculated using ANOVA with Tukey’s multiple-comparison test. Data are presented as the mean ± S.D. **h** μCT images of the bone formed by PSCs derived from *Prmt5*^flox/Δ^ *Ctsk*-Cre CAG-CAT-EGFP and *Prmt5*^flox/+^ *Ctsk*-Cre CAG-CAT-EGFP. Representative images of more than three independent experiments (left) and quantification of bone mineral content (right) are shown (*n* = 7 and *n* = 5 mice per group). Scale bars, 5 mm. *P* value was calculated using one-sided Student’s *t*-test. Data are presented as the mean ± S.D. **i** Representative images from more than three independent Von Kossa staining (black) for mineralized bone formed by PSCs derived from *Prmt5*^flox/Δ^ *Ctsk*-Cre CAG-CAT-EGFP and *Prmt5*^flox/+^ *Ctsk*-Cre CAG-CAT-EGFP mice. Scale bars, 200 μm. Black arrow heads indicate mineralized bone. Source data are provided as a Source Data file.

In order to elucidate the role and target of Prmt5 in PSCs, we performed RNA-seq analysis on PSCs collected from *Prmt5*^flox/Δ^ *Ctsk*-Cre CAG-CAT-EGFP mice or *Prmt5*^flox/+^ *Ctsk*-Cre CAG-CAT-EGFP mice. The RNA-seq data showed the gene expression pattern in *Prmt5*-deficient PSCs was very different from that in control PSCs (Fig. 3a, b). We found that the mRNA expression level of *Postn* (encoding periostin) was markedly decreased in Prmt5-deficient PSCs (Fig. 3c, d). We focused on periostin because periostin is known to be required for the self-renewal of osteogenic progenitors in the periosteum^20,21^. Previous studies have indicated that the primary role of Prmt5 is to regulate pre-mRNA splicing by mediating symmetrical demethylation of arginine residues in Sm proteins^12,13^. The mis-splicing of pre-mRNA due to Prmt5 deficiency can cause downregulation of mRNA expression by the nonsense-mediated mRNA decay mechanism^12,13^. RNA-seq analysis indicated that an abnormal splicing of *Postn* mRNA occurred in *Prmt5*-deficient PSCs (Fig. 3e). Immunohistochemistry confirmed the decreased POSTN protein levels in the periosteum in *Prmt5*^flox/Δ^ *Ctsk*-Cre CAG-CAT-EGFP mice (Fig. 3f and Supplementary Fig. 7i). The localization of EGFP^+^ POSTN^+^ cells adjacent to the growth plate is consistent with a previous report which identified PSCs^3^.

Similar to the finding in *Postn*-deficient periosteal osteogenic progenitors^21^, the colony forming ability was impaired in *Prmt5*-deficient PSCs (Fig. 3g). Treatment with exogenous periostin partially rescued the impaired self-renewal ability in *Prmt5*-deficient PSCs (Fig. 3g). In vivo transplantation experiments showed that PSCs derived from control mice exclusively formed intramembranous bones (Fig. 3h, i), as reported in a previous study^3^. In contrast, Prmt5-deficient PSCs formed minimal intramembranous bones but not cartilage or adipose tissue (Fig. 3h, i). These data suggested that the Prmt5/periostin axis is important for self-renewal of PSCs, but further studies are needed to clarify the role of Prmt5 in the later differentiation and lineage commitment stages in PSC-mediated bone formation. The mRNA expression levels of marker genes for periosteal osteogenic progenitors^3,22–24^ and skeletal stem cells^5^ did not change in Prmt5-deficient PSCs, suggesting that Prmt5 is important for the self-renewal but not for stem cell identity in PSCs (Supplementary Fig. 7j). These data suggest that abnormal post transcriptional regulation of *Postn* may cause, at least in part, a progressive reduction in the PSC number in *Prmt5*^flox/Δ^ *Ctsk*-Cre mice. This hypothesis is consistent with a previous study showing that periostin-deficient mice exhibited postnatal defects in endochondral and intramembranous bone formation^25^.

### PSCs orchestrate both types of bone growth via Ihh secretion

How do PSCs, located in the periosteum/perichondrium, control endochondral bone formation at the growth plate? A previous study concluded that PSCs’ role is exclusively limited to intramembranous bone formation under physiological conditions for the following reasons. First, *Ctsk*-Cre labels periosteal osteogenic cells, but not growth plate chondrocytes or marrow osteoblasts in a fate-mapping system, indicating that PSCs do not act as a source for the osteogenic cells that mediate endochondral bone formation^3^. Second, PSCs form bone via an intramembranous pathway in a transplantation system^3^. And third, *Ctsk*-Cre-mediated deletion of *Sp7* results in bone loss in intramembranous bones such as the calvarium and cortical bone, but does not affect growth plate architecture or trabecular bone^3^. Because this is in contrast with our phenotype, where the growth plate is markedly altered during postnatal growth in PSC-deficient mice, we tested the hypothesis that PSCs could contribute indirectly to endochondral bone formation by locally producing soluble factors.

**Fig. 4:**
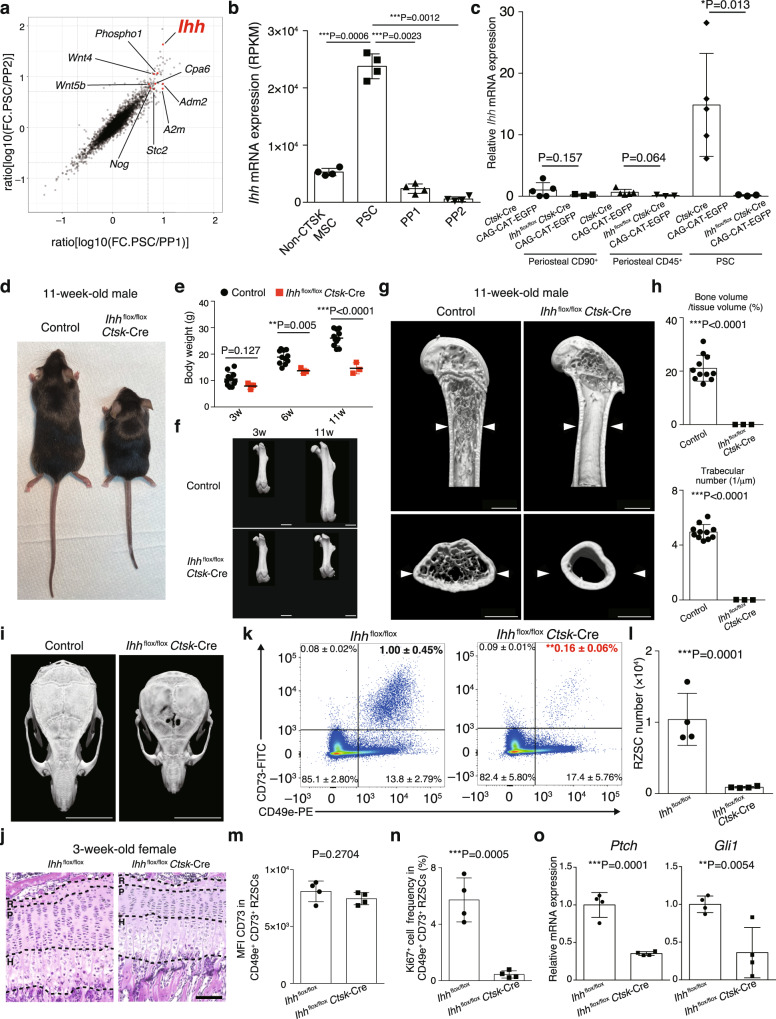
PSC-derived Ihh orchestrates bone growth. **a** Scatter plot of the gene expression ratio. **b** *Ihh* expression in periosteal osteogenic progenitors and CTSK^–^ bone marrow mesenchymal stem cells (*n* = 4 biologically independent samples). *P* values were calculated using ANOVA with Tukey’s multiple-comparison test. Data are presented as the mean ± S.D. **c** *Ihh* expression in various periosteal cell types (*n* = 5 and 3 mice per group). *P* values were calculated using one-sided Student’s *t*-test. Data are presented as the mean ± S.D. **d** Images of littermates. *Ihh*^flox/+^, *Ihh*^flox/flox^, *Ihh*^flox/+^ *Ctsk*-Cre mice were phenotypically identical, being grouped together and used as controls in (**d**–**h**). **e** Body weight of littermates (*n* = 11 and 3 mice per group). *P* values were calculated using one-sided Student’s *t*-test. Data are presented as the mean ± S.D. **f** Representative femur images of more than three littermates. Scale bars, 2 mm. **g** Representative femur images analyzed in **h**. Scale bars, 1 mm. The white arrow heads indicate bone width in the control mice. **h** μCT analysis parameters of the femur in littermates (*n* = 11 and 3 mice per group). *P* values were calculated using one-sided Student’s *t*-test. Data are presented as the mean ± S.D. **i** Representative skull images of more than three female littermates. Scale bars, 7 mm. **j** Representative haematoxylin and eosin staining pictures of the tibiae growth plate in more than three littermates. Scale bars, 100 μm. R: resting zone, P: proliferating zone, H: hypertrophic zone. **k** Frequency of RZSCs in 3-week-old littermates (*n* = 4 mice per group). *P* = 0.0051. *P* value was calculated using one-sided Student’s *t*-test. **l** RZSC number in 3-week-old littermates (*n* = 4 mice per group). *P* value was calculated using one-sided Student’s *t*-test. Data are presented as the mean ± S.D. **m** Mean Fluorescence Intensity of CD73 in RZSCs (*n* = 4 mice per group). *P* value was calculated using one-sided Student’s *t*-test. Data are presented as the mean ± S.D. **n** Ki67^+^ cell frequency in RZSCs in littermates (*n* = 4 mice per group). *P* value was calculated using one-sided Student’s *t*-test. Data are presented as the mean ± S.D. **o** The expression levels of *Ptch* and *Gli1* in RZSCs (*n* = 4 mice per group). *P* values were calculated using one-sided Student’s *t*-test. Data are presented as the mean ± S.D. Source data are provided as a Source Data file.

We therefore screened for genes that are highly and specifically expressed in PSCs by analyzing a deposited RNA-seq dataset (GSE106237)^3^. We focused on soluble factors having much higher expression in PSCs than PP2 and PP1. Ihh was among the genes highly specific to PSCs and is known to be involved in the regulation of endochondral bone formation (Fig. 4a). The major producer of Ihh has been thought to be hypertrophic chondrocytes based on the in situ hybridization experiments using fetal bones, but the contribution of Ihh produced by other cells has never been reportedly explored^6,7,9–11,26,27^. More relevant to our observations is the fact that among the periosteal osteogenic progenitors (PP1, PP2 and PSC) and CTSK^–^ bone marrow mesenchymal stem cells, Ihh is exclusively expressed in PSCs (Fig. 4b).

We therefore crossed *Ihh*^flox/flox^ mice^7^ with *Ctsk*-Cre mice to investigate the physiological relevance of Ihh produced by PSCs. In *Ihh*^flox/flox^ *Ctsk*-Cre CAG-CAT-EGFP mice, the expression levels of Ihh decreased specifically in PSCs, but not in the growth plate chondrocytes, periosteal CD90^+^ mature osteogenic cells or periosteal CD45^+^ hematopoietic cells (Fig. 4c and Supplementary Fig. 8a, b). Since Ihh is known to be expressed in hypertrophic chondrocytes, we rigorously excluded the possibility that *Ctsk*-Cre leaked in hypertrophic chondrocytes by using RANKL-floxed mice^28^. RANKL (encoded by *Tnfsf11* gene), the master regulator of osteoclastogenesis, is essentially produced by hypertrophic chondrocytes, osteoblasts and osteocytes^28,29^. The deletion of RANKL in hypertrophic chondrocytes by *Col10a1*-Cre^30^ resulted in severe osteopetrosis due to a reduction of osteoclast number in the bone marrow (Supplementary Fig. 8c–e, g). In contrast, *Tnfsf11*^flox/flox^ *Ctsk*-Cre mice did not exhibit osteopetrosis and a decrease in osteoclast number in these mice was observed only at the periosteal surface but not in the bone marrow (Supplementary Fig. 8f). If *Ctsk*-Cre deleted RANKL in hypertrophic chondrocytes, marrow osteoblasts or osteocytes, *Tnfsf11*^flox/flox^ *Ctsk*-Cre mice would have exhibited severe osteopetrosis. These data clearly indicated that *Ctsk*-Cre does not leak in hypertrophic chondrocytes, marrow osteoblasts or osteocytes.

Notably, *Ihh*^flox/flox^ *Ctsk*-Cre mice displayed a severe reduction in body size, body weight and bone length, indicating that endochondral bone formation was impaired (Fig. 4d–h). This phenotype became more readily apparent with aging (Fig. 4e, f), and *Ihh*^flox/flox^ *Ctsk*-Cre mice showed a complete loss of trabecular bone and the growth plate at the age of 11 weeks (Fig. 4g, h). *Ihh*^flox/flox^ *Ctsk*-Cre mice exhibited a marked reduction in bone width and calvarial bone volume (Fig. 4g, i), indicating that PSC-derived Ihh is also required for intramembranous bone formation. Bone marrow transfer experiments confirmed that Ctsk^+^ mesenchymal cells, but not hematopoietic cells, were responsible for the skeletal phenotype in *Ihh*^flox/flox^ *Ctsk*-Cre mice (Supplementary Fig. 9a–d). *Ihh*^flox/flox^ *LysM*-Cre mice displayed normal bone growth, indicating that the deletion of Ihh in osteoclast-lineage cells does not contribute to the skeletal phenotype in *Ihh*^flox/flox^ *Ctsk*-Cre mice (Supplementary Fig. 9e–j).

### PSC-derived Ihh is required for RZSC maintenance

At 3 weeks of age, *Ihh*^flox/flox^ *Ctsk*-Cre mice retained normal growth plate architecture (Fig. 4j). Since Ihh is known to be a key regulator of RZSCs in the growth plate, we analyzed the CD73^+^ CD49e^+^ RZSCs^2^ in *Ihh*^flox/flox^ *Ctsk*-Cre mice. Flow cytometry showed that the frequency and number of CD73^+^ CD49e^+^ RZSCs markedly decreased in the growth plate of *Ihh*^flox/flox^ *Ctsk*-Cre mice (Fig. 4k, l). The expression levels of stem cell marker CD73 in RZSCs was unchanged (Fig. 4m), whereas the frequency of Ki67^+^ cells in RZSCs significantly decreased in *Ihh*^flox/flox^ *Ctsk*-Cre mice (Fig. 4n). These data are consistent with a previous report showing that Ihh is critical for the self-renewal of RZSCs but not for their identity^2^. Given that 3-week-old *Ihh*^flox/flox^ *Ctsk*-Cre mice did not display obvious bone abnormalities other than the reduction of RZSCs, it is likely that RZSCs are the direct target of PSC-derived Ihh. Indeed, the expression levels of Ihh target genes, *Ptch* and *Gli1*, were significantly decreased in RZSCs collected from *Ihh*^flox/flox^ *Ctsk*-Cre mice (Fig. 4o).

To clarify the relative contribution between Ihh derived from PSCs and from hypertrophic chondrocytes, we crossed *Ihh*^flox/flox^ mice with *Col10a1*-Cre mice. *Ihh*^flox/flox^ *Col10a1*-Cre mice had already exhibited severe bone deformities with an abnormal growth plate architecture at 3 weeks of age (Supplementary Fig. 10a–c). These data indicate that hypertrophic chondrocyte-derived Ihh is essential and sufficient for bone development at the early stages of life, whereas the importance of PSC-derived Ihh increases over time in postnatal life. Since *Ihh*^flox/flox^ *Ctsk*-Cre mice displayed a defect in intramembranous bone formation (Fig. 4g, i), we analyzed the PSC phenotypes in these animals and found that the frequency and number of PSC decreased in *Ihh*^flox/flox^ *Ctsk*-Cre CAG-CAT-EGFP mice (Supplementary Fig. 10d, e). These data suggested that PSC-derived Ihh may also be important for the maintenance of PSC itself, but further studies are required to understand the role of PSC-derived Ihh in the periosteum homeostasis.

Collectively, these findings demonstrate that as Ihh production by growth plate chondrocytes decreases during the progressive decline in growth with age, Ihh production by periosteal PSCs maintains RZSC proliferation in the growth plate, thereby playing a key role in the prolonged postnatal bone growth.

## Discussion

This study shows that although PSCs are specialized into creating osteogenic progenitors for the intramembranous pathway that occurs on the periosteal surface, they are also essential for the prolonged endochondral bone formation that occurs in the growth plate via their ability to locally produce Ihh. It is well established that Ihh plays an essential role in endochondral bone formation at the growth plate by regulating the fate of RZSCs^1,2^. During development, growth plate-derived Ihh acts on cells in the periosteum/perichondrium, leading to the activation of PTHrP expression in the periarticular chondrocytes through a poorly understood mechanism^10,11^. PTHrP then maintains chondrocytes in a proliferative, less differentiated state and inhibits the production of Ihh from the growth plate. This Ihh/PTHrP loop coordinates the synchronized chondrocyte differentiation in the growth plate during early life stages^6^.

Here, we show a reverse crosstalk that occurs later, at a time when the growth plate decreases its proliferative activity and growth speed in postnatal life. PSC-derived Ihh controls growth plate RZSCs to ensure prolonged skeletal growth in postnatal life. This study therefore highlights the critical importance of a functional interaction between PSCs and RZSCs in post-developmental skeletal growth. This view is supported by the fact that *Ihh*^flox/flox^ *Ctsk*-Cre mice displayed a severe defect in bone growth at the adult stage, but the phenotype was not apparent before 3 weeks of age (Fig. 4 and Supplementary Fig. 10). Given that the width of the hypertrophic zone starts to regress around 3 weeks of age^31^, we interpret this data to show that hypertrophic chondrocyte-derived Ihh is sufficient to ensure rapid bone growth during development and early postnatal life, whereas PSC-derived Ihh compensates for the progressive decrease in hedgehog signaling in RZSCs during postnatal life, ensuring continued bone growth.

We analyzed PSCs using the same *Ctsk*-Cre line and cell surface markers with a previous report which first identified and characterized PSCs^3^. Using the RNAseq dataset (GSE106237) produced by the study^3^, we identified the exclusive Ihh expression in PSCs among periosteal osteogenic cells (Fig. 4a, b). Thus, the PSC analyzed here is exactly the same population with the previous study^3^. Given that the PSCs are localized to adjacent to the growth plate^3,15^ (Fig. 3f), we suspect that PSC-derived Ihh may control RZSCs in a paracrine manner. A previous study showed that diffusion of Shh from the secondary ossification center (SOC) also contributes to the growth plate homeostasis^2^. Thus, vertical (from the SOC and hypertrophic zone) and horizontal (from the periosteum/perichondrium) regulation of hedgehog signaling in the growth plate may be required for the precise coordination of skeletal growth.

Intramembranous and endochondral bone formation have been considered to be independent processes. However, our data reveals that intramembranous and endochondral pathways cooperatively ensure proper development and maintenance of bone growth for a sufficient period of time to allow long bones to reach their adult size and shape. During fracture repair, PSCs were shown to undergo endochondral bone formation in response to bone injury due to an unknown mechanism^3^. We suspect that PSC-derived Ihh may also play a role in the context of fracture healing. Further studies are required to clarify the relevance and function of PSC-derived Ihh in fracture repair. Given that early vertebrates first acquired intramembranous pathway to form exoskeleton (dermal bone)^32,33^, we suspect that PSCs may have developed earlier than other types of skeletal stem cells, subsequently acquiring the capacity to regulate endochondral bone formation.

Collaborations between distinct types of stem cells have been previously documented in studies of *Drosophila* gonads^34,35^ and mammalian skin^36^. This study shows the importance of stem cell communication in bone growth and homeostasis, suggesting that the interactions that occur between and among stem cells may be a fundamental principle in the development and maintenance of mammalian organs in general. Investigation into the molecular mechanisms of the functional crosstalk between stem cells will be a key to ultimately understanding the physiology and pathology of complicated biological systems.

## Methods

### Mice and bone analysis

All animals were maintained under specific pathogen-free conditions, and all experiments were performed with the approval of the Institutional Review Board at The University of Tokyo. C57BL/6 mice were purchased from CLEA Japan. KSN/Slc Nude mice (6-8 week-old male) were purchased from SLC Japan. *R26*-*iDTR* mice, *Ihh*^flox/flox^ mice and B6.SJL (CD45.1^+^) mice were obtained from the Jackson Laboratory. *Prmt5*^flox/flox^ mice^12^, *Ctsk*-Cre mice^14^, CAG-CAT-EGFP mice^37^, *Col2a1*-Cre mice^18^, *Sp7*-Cre mice^19^, *LysM*-Cre mice^17^, *Col10a1*-Cre mice^30^ and *Tnfsf11*^flox/flox^ mice^28^ were described previously. Three-dimensional microcomputed tomography analyses and bone morphometric analyses were performed as described^38^. Age and sex-matched littermates were used for all of the experiments unless otherwise noted. The sex and age of mice used are described in figures or figure legends. All animals were maintained at a constant ambient temperature of 22–26 degree Celsius, 40–65% of humidity under a 12 h light/dark cycle with free access to food and drink.

### Cell isolation

For the isolation of periosteal cells, long bones without muscles were subjected to enzymatic digestion for 1 h with serum-free α-MEM medium containing Collagenase (1 mg/ml; Wako, cat. 032-22364), Dispase II (2 mg/ml; Roche, cat. 383-02281) and DNase I (1 mg/ml; SIGMA, cat. DN25-1G) at 37 °C with agitation. After the removal of the long bones without periosteum, the tubes were centrifuged to harvest periosteal cells as pellet cells. α-MEM medium containing 20% serum was added to the tube and the periosteal cells were resuspended thoroughly by pipetting and then filtered through 100-μm nylon mesh. Tubes were centrifuged and the resulting cell pellet was subjected to FACS using the gating strategy shown in Supplementary Fig. 6. For the isolation of growth-plate chondrocytes, a scalpel was used to cut between the hypertrophic zone and the primary spongiosa of the long bones. Then, the blunt side of the scalpel was used to carefully scrape out the cartilage until the blade reached the hard surface of the secondary ossification center. The pieces of cartilage were collected and treated with 0.15% collagenase II in serum-free α-MEM medium for 120 min at 37 °C with agitation. The collected growth plate cells were subjected to FACS and western blotting analyses. The absolute number of PSCs and RZSCs per two legs was measured by using FACSAria III (BD Biosciences). We observed higher numbers/percentages of PSCs than that in the previous report^3^. This may reflect that we performed a whole-bone periosteal digest whereas the previous study largely minced entire bones from younger mice^3^. We set the CD200 and CD105 gates based on the FACS plot of negative controls (non-staining periosteal cell) (Supplementary Fig. 10d).

### RNA-seq analysis and quantitative RT-PCR analysis

Bulk-RNA sequencing and real-time quantitative PCR with reverse transcription (RT-PCR) analyses were performed as described^12^. In brief, total RNA was extracted using the Maxwell 16 LEV simply RNA Tissue Kit (Promega) according to the manufacturer’s instructions. cDNA was synthesized using Superscript III reverse transcriptase and a SMART-seq v4 Ultra Low Input RNA Kit for Sequencing (Clontech Laboratories) for RNA-seq and RT-PCR, respectively. In the RNA-seq analysis, cDNA and sequencing libraries were processed with FastQC (v0.1.1.8), TrimGalore (v0.6.4) and Kallisto (v0.46.0) using the mouse transcriptome index (mus musculus GRCm38.96), and analyzed by DESeq2 (v1.26) to quantify gene expression on the basis of PCA, hierarchical clustering and differential expression analysis (using log fold change shrinkage and an adjusted *p* value <0.05). For the analysis on bulk-RNA seq data of periosteal progenitors (GSE106235), scatter plot of the gene expression ratio between PSC/PP1 and PSC/PP2 was produced. The soluble factors highly and specifically expressed in PSCs (RPKM > 500 in PSCs, PSC/PP1 ratio >5, PSC/PP2 ratio >5 and RPKM > 0 in PSC, PP1 and PP2) are highlighted in the Fig. 4a. RT-PCR analysis was performed with a LightCycler (Roche) using SYBR Green (Toyobo) and the level of mRNA expression was normalized to *Gapdh*. All of the primer sequences are available upon request.

### Flow cytometry and antibodies

The monoclonal antibodies used were purchased from BioLegend and eBioscience. For periosteal osteogenic progenitor analysis, cells were stained with PE anti-mouse CD51 (RMV-7), APC anti-mouse CD200 (OX-90), PB anti-mouse CD90.2 (53-2.1), PerCP-Cy5.5 anti-mouse CD105 (MJ7/18), APCcy7 anti-mouse CD45 (30-F11), biotinylated anti-mouse Ter119 (TER-119), biotinylated anti-mouse CD31 (390), biotinylated anti-mouse Ly-51 (6C3), and PEcy7 streptavidin (B278254). For analysis on resting zone stem cells, FITC anti-mouse CD73 (TY/11.8), PE anti-mouse CD49e (MFR5) and APC anti-mouse Ki67 (16A8) antibodies were used. The Foxp3 Staining Buffer Set (eBioscience) was used for intracellular Ki67 staining. For haematopoietic stem and progenitor cell analysis, cells were stained with PE anti-mouse Flk2 (A2F10), APC anti-mouse Sca-1 (D7), eFluor 450 anti-mouse CD34 (RAM34), PerCP-Cy5.5 anti-mouse CD150 (mShad150), PE-Cy7 anti-mouse CD48 (HM48-1), APCcy7 anti-mouse CD117/c-kit (2B8), FITC streptavidin (eBioscience), PE anti-mouse CD16/32 (93), PE anti-mouse Ter119 (TER-119), FITC anti-mouse CD71 (RI7217) and biotinylated anti-mouse CD3ε (145-2C11), CD4 (RM4-5), CD8a (53-6.7), CD11b (M1/70), CD11c (HL3), CD45R/B220 (RA3-6B2), TER119 (TER-119), Gr-1 (RB6-8C5), and CD49b (DX5). LT-HSCs, GMPs, CMPs, MEPs and Pro-E were defined as Lin^–^Sca1^+^c-Kit^+^CD150^+^CD34^−^CD48^−^Flk2^−^ cells, Lin^−^Sca1^+^c-Kit^+^CD34^+^CD16/32^hi^ cells, Lin^–^Sca1^+^c-Kit^+^CD34^+^CD16/32^mid^ cells, Lin^–^Sca1^+^c-Kit^+^CD34^−^CD16/32^−^ cells and c-Kit^+^CD71^+^Ter119^lo^ cells, respectively^39,40^. FITC anti-mouse CD45.1 (A20) and PE anti-mouse CD45.2 (104) antibodies were used to examine bone marrow chimerism. Flow cytometric analysis and sorting were performed using FACSCanto II and FACSAria III (BD Biosciences). PerCP-Cy5.5 anti-mouse CD105 antibody was used at 1:50 dilution and other antibodies were used at 1:100 dilution. FlowJo V9.9.3 software (TreeStar) was used to analyze the FACS data.

### Immunohistochemistry

Femurs and tibiae were fixed overnight at 4 °C in 4% paraformaldehyde for cryosection. Samples were washed twice in PBS and then embedded in OCT compound (Sakura Finetek). The frozen tissue blocks were cut into 20-μm-thick sections using a Cryostat (Leica). The sections were stained with anti-mouse periostin (Abcam, ab14041, 1:100 dilution), anti-GFP (invitrogen, A10262, 1:100 dilution) and DAPI (Molecular Probes). Multicolor images were obtained with a Nikon C2 confocal microscope (Nikon).

### Western blot analysis

The PSCs and growth plate chondrocytes were lysed with RIPA buffer (nacalai tesque) containing protease inhibitor cocktail (Millipore Sigma). The lysate was mixed with Trident 4X Laemmli SDS Sample Buffer containing 8% 2-mercaptoethanol, then boiled at 95 °C for 5 mins. The proteins were subjected to SDS-PAGE (5-20% gradient gel, FUJIFILM) and transferred onto a PVDF membrane (Millipore). The antibodies used were as follows: anti-β-actin (A5441, 1:1000 dilution), Sigma-Aldrich; anti-PRMT5 (PRMT5-21, 1:200 dilution), Santa Cruz; anti-IHH (ab52919, 1:200 dilution), Abcam.

### In situ hybridization

The in situ hybridization experiments were outsourced to Genostaff Co. Ltd. In brief, bone tissues were fixed with G-Fix (Genostaff), de-calcified with G-Chelate Mild (Genostaff), embedded in paraffin on a CT-Pro20 (Genostaff) using G-Nox (Genostaff) as a less toxic organic solvent for xylene, and sectioned at 5–8 μm. In situ hybridization was performed with the ISH Reagent Kit (Genostaff) according to the manufacturer’s instructions. Tissue sections were de-paraffined with G-Nox, and rehydrated through an ethanol series and phosphate-buffered saline (PBS). The sections were fixed with 10% NBF (10% formalin in PBS) for 30 min at 37 °C and washed in distilled water, placed in 0.2 N HCl for 10 min at 37 °C and washed in PBS, treated with 4 μg ml^−1^ ProteinaseK (Wako Pure Chemical Industries) in PBS for 10 min at 37 °C and washed in PBS, then placed within a coplin jar containing 1xG-Wash (Genostaff), equal to 1xSSC. Hybridization was performed with probes at concentrations of 250 ng ml^−1^ in G-Hybo-L (Genostaff) for 16 h at 60 °C. After hybridization, the sections were washed in 1xG-Wash for 10 min at 60 °C, 50% formamide in 1xG-Wash for 10 min at 60 °C. Then the sections were washed twice in 1xG-Wash for 10 min at 60 °C, twice in 0.1xG-Wash for 10 min at 60 °C and twice in TBST (0.1% Tween 20 in tris-buffered saline (TBS)) at room temperature (RT). After treatment with 1xG-Block (Genostaff) for 15 min at RT, the sections were incubated with anti-DIG AP conjugate (Roche Diagnostics) diluted 1:2000 with x50G-Block (Genostaff) in TBST for 1 h at RT. The sections were washed twice in TBST and then incubated in 100 mM NaCl, 50 mM MgCl_2_, 0.1% Tween 20, 100 mM Tris-HCl, pH 9.5. Coloring reactions were performed with NBT/BCIP solution (Sigma-Aldrich) overnight and then the samples were washed in PBS. The sections were counterstained with Kernechtrot stain solution (Muto Pure Chemicals), and mounted with G-Mount (Genostaff). The probes for *Ihh* was developed by Genostaff.

### Bone marrow transfer

Single-cell suspensions were obtained from the bone marrow of donor mice. Recipient mice were sub-lethally irradiated and administered an injection of bone marrow cells (2 × 10^6^ cells). Mice were provided antibiotic-containing water for two weeks and analyzed eight weeks later. Since the irradiation affects bone turnover and decreases baseline bone volume, control mice were also sub-lethally irradiated and subjected to the same experimental procedure in all bone marrow transfer experiments. Bone marrow chimerism was analyzed by FACS using B6.SJL (CD45.1^+^) mice and antibodies, FITC anti-mouse CD45.1 (A20) and PE anti-mouse CD45.2 (104). The bone marrow transfer system used in this study efficiently replaced bone marrow haematopoietic cells (>95% engraftment).

### Kidney capsule transplantation

Six to eight-week-old male KSN/Slc Nude mice were anaesthetized. The kidney was externalized through a 1-cm incision and a 2-mm pocket was made in the renal capsule. A 5-µl Matrigel plug (Corning, 356231) containing 10,000 cells was implanted underneath the capsule and kidney was replaced back into the body cavity. Animals were euthanized after six weeks. After death, kidneys were fixed overnight with 4% PFA and bone formation was detected by μCT. Samples were subjected to infiltration, embedding and sectioning. Von Kossa staining was performed to detect the mineralized bones.

### Cell culture

For in vitro osteoclastogenesis, primary bone marrow cells were cultured with M-CSF (R&D Systems) for two days. These cells were further cultured for three or five days with RANKL (Wako) in the presence of M-CSF, as previously reported^41^. The culture medium was changed every second day in all of the experiments. Osteoclastogenesis was evaluated by counting TRAP-positive multinucleated (more than three nuclei) cells and the bone resorbing activity was confirmed by analyzing the resorption area after staining the bone slices with 0.5% toluidine blue. For the colony-forming efficiency assay, primary PSCs were sorted and plated on 12-well pates at a concentration of 500 cells/well in culture medium (DMEM with 10% FBS) in the presence or absence of 200 ng/ml recombinant mouse periostin (2955-F2-050, R&D) for 14 days. Cells were cultured on the FALCON^®^ tissue culture plate (tissue culture treated by vacuum gas plasma) at a constant ambient temperature of 37 degree Celsius and 5.0% CO_2_. We used α−MEM (Gibco, Thermo Fisher Scientific, Waltham, MA, USA) and DMEM (1.0 g/l Glucose with L-Gln and Sodium Pyruvate liquid, Nacalai Tesque) for culture of osteoclasts and PSCs, respectively.

### Statistical analyses

Data were analyzed on GraphPad Prism software version 6.0 g. Statistical tests, n values, replicate experiments, and *P* values are all located in the figures and/or legends. All data are presented as the mean ± S.D. *P* values were calculated using Student’s *t*-test and analysis of variance (ANOVA) with Dunnett’s or Tukey’s multiple-comparison test.

### Reporting summary

Further information on research design is available in the Nature Research Reporting Summary linked to this article.

## Supplementary information

Supplementary Information

Reporting Summary

## Data Availability

The data that support the plots within this paper and other findings of this study are available from the corresponding author upon reasonable request. The RNAseq data produced in this study were deposited to the public data base GSE146872. We also used the bulk-RNA seq data for periosteal progenitors GSE106235 and the mouse transcriptome index mus musculus GRCm38.96 [https://www.ncbi.nlm.nih.gov/assembly/GCF_000001635.20/]. Source data are provided with this paper.

## Source data

Source Data
