# Supplementary material for: Periosteal stem cells control growth plate stem cells during postnatal skeletal growth
Source: Nat Commun. 2022 Jul 18;13:4166. doi: 10.1038/s41467-022-31592-x (PMC9293991; doi:10.1038/s41467-022-31592-x)
Supplement: Supplementary file 1 — Supplementary Information [file 41467_2022_31592_MOESM1_ESM.pdf]

Supplementary Information

Periosteal stem cells control growth plate stem cells during postnatal skeletal growth

Tsukasaki et al.

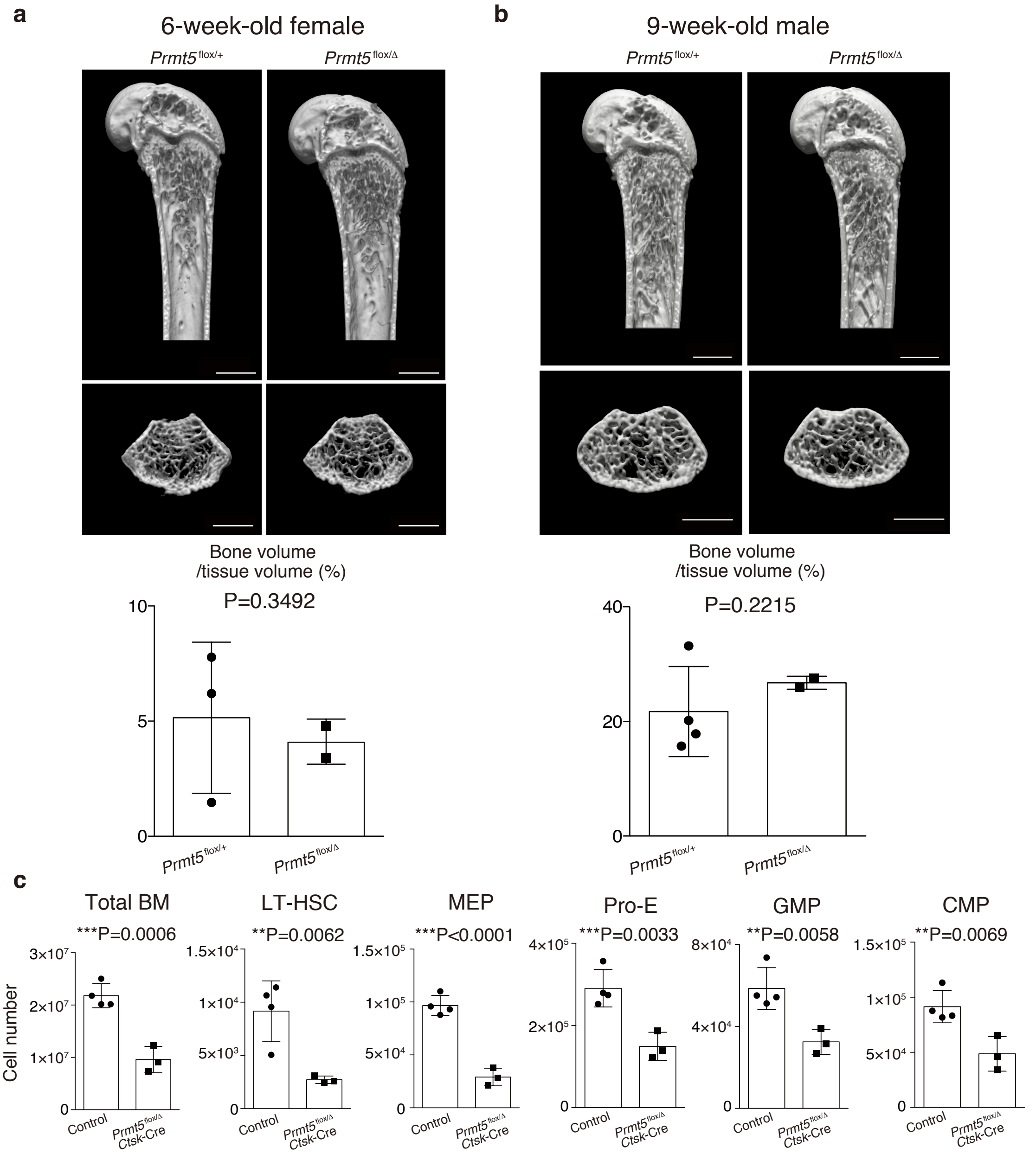

Supplementary Figure 1. Normal bone phenotypes in *Prmt5*$^{\text{flox/}\Delta}$ mice and the decreased bone marrow cellularity in aged *Prmt5*$^{\text{flox/}\Delta}$ *Ctsk*-Cre mice. a, Representative μCT images (upper panels) of more than two independent experiments and parameters of μCT analysis (lower panel) of the femur in 6-week-old female *Prmt5*$^{\text{flox/+}}$ and *Prmt5*$^{\text{flox/}\Delta}$ mice (n=3 and 2 mice per group). Scale bars, 1 mm. *P* value was calculated using one-sided Student's *t*-test. Data are presented as the mean ± S.D. **b,** Representative μCT images more than two independent experiments (upper panels) and parameters of μCT analysis (lower panel) of the femur in 9-week-old male *Prmt5*$^{\text{flox/+}}$ and *Prmt5*$^{\text{flox/}\Delta}$ mice (n=4 and 2 mice per group). Scale bars, 1 mm. *P* value was calculated using one-sided Student's *t*-test. Data are presented as the mean ± S.D. **c,** Total haematopoietic cell counts and the numbers of LT-HSC (long

term-haematopoietic stem cell), MEP (megakaryocytic erythroid progenitors), Pro-E (pro-erythroblasts), GMP (granulocyte-macrophage progenitors) and CMP (common myeloid progenitor) per femur in male control and *Prmt5*$^{flox/\Delta}$ *Ctsk*-Cre mice at 40-50 weeks of age (n=4 and 3 mice per group). *P* values were calculated using one-sided Student's *t*-test. Data are presented as the mean ± S.D. Source data are provided as a Source Data file.

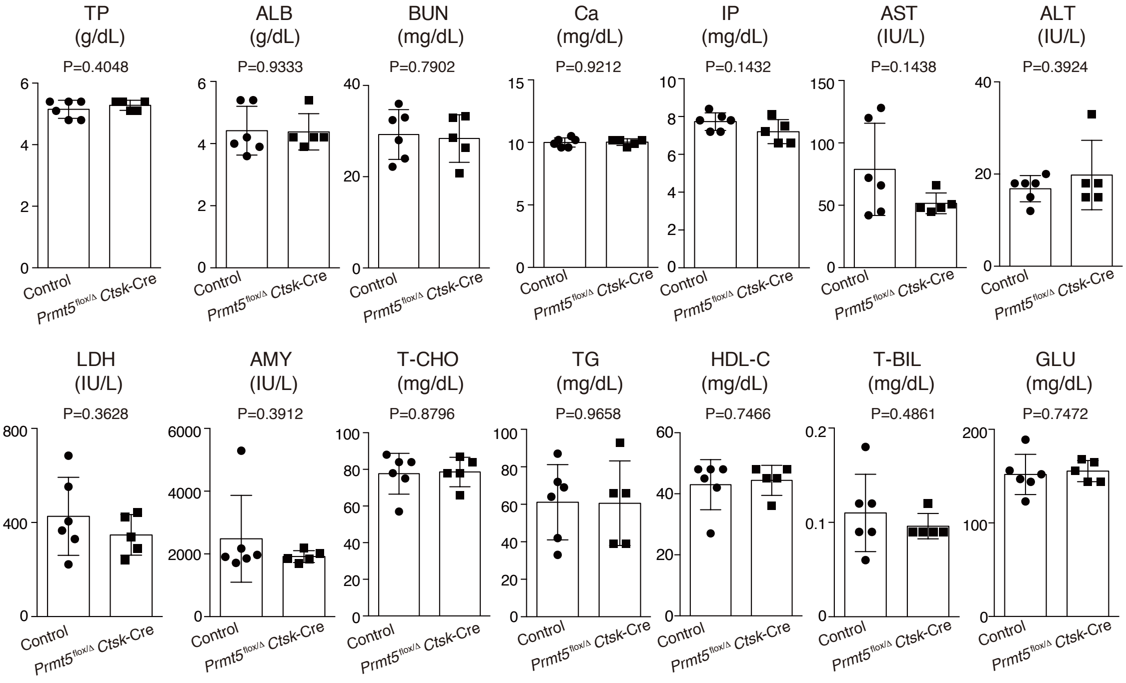

Supplementary Figure 2. The results of blood-screening in control and *Prmt5*$^{\text{flox}/\Delta}$ *Ctsk*-Cre mice. The serum concentration of the total protein (TP), albumin (ALB), blood urea nitrogen (BUN), calcium (Ca), inorganic phosphate (IP), aspartate aminotransferase (AST), alanine aminotransferase (ALT), lactate dehydrogenase (LDH), amylase (AMY), total cholesterol (T-CHO), triglyceride (TG), high-density lipoprotein cholesterol (HDL-C), total bilirubin (T-BIL) and glucose (GLU) in male control and *Prmt5*$^{\text{flox}/\Delta}$ *Ctsk*-Cre mice at 11 weeks of age (n=6 and 5 mice per group). *Prmt5*$^{\text{flox}/+}$, *Prmt5*$^{\text{flox}/\Delta}$, and *Prmt5*$^{\text{flox}/+}$ *Ctsk*-Cre mice were grouped together and used as controls. The nutritional status, the function of kidney, liver, pancreas and heart, and metabolism of fat and sugar are not altered in *Prmt5*$^{\text{flox}/\Delta}$ *Ctsk*-Cre mice, indicating that the skeletal phenotypes of *Prmt5*$^{\text{flox}/\Delta}$ *Ctsk*-Cre mice are not attributed to a failure to thrive caused by ectopic expression of *Ctsk*-Cre in other organs. *P* values were calculated using one-sided Student's *t*-test. Data are presented as the mean ± S.D. Source data are provided as a Source Data file.

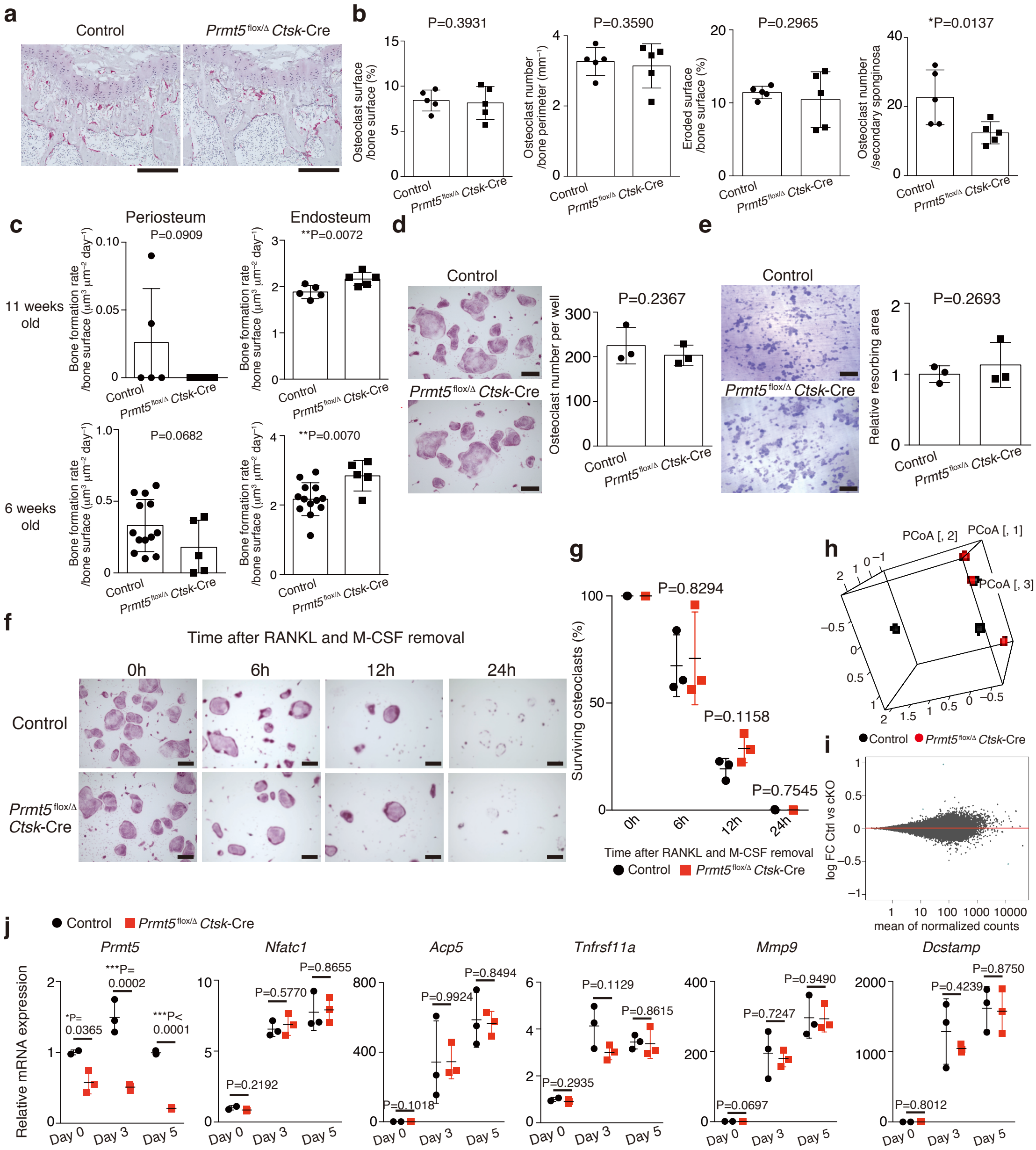

Supplementary Figure 3. Prmt5-deficiency doses not affect osteoclasts. a, TRAP staining of the proximal tibiae of 11-week-old female littermates. Representative pictures of more than three independent experiments are shown. Scale bars, 200μm. **b,** Osteoclastic parameters measured by bone morphometric analysis in 11-week-old female littermates (n=5 mice per group). The total osteoclast number in the secondary sponginosa decreased in *Prmt5*$^{flox/\Delta}$ *Ctsk*-Cre mice due to a low bone mass and a reduced haematopietic progenitor cells, but the osteoclastic parameters normalized by bone surface and bone perimeter indicated that osteoclast differentiation was not altered in these animals. *P* values were calculated using one-sided Student's *t*-test. Data are presented as the mean ± S.D. **c,** Bone formation rate per bone surface in the periosteum and endosteum in 11-week-old (n=5 mice per group) and 6-week-old (n=13 and 5 mice

per group) littermates. *P* values were calculated using one-sided Student's *t*-test. Data are presented as the mean ± S.D. **d,** *In vitro* osteoclast differentiation (n=3 mice per group). Scale bars, 200μm. *P* value was calculated using one-sided Student's *t*-test. Data are presented as the mean ± S.D. **e,** *In vitro* osteoclastic bone resorption (n=3 mice per group). Scale bars, 200μm. *P* value was calculated using one-sided Student's *t*-test. Data are presented as the mean ± S.D. **f,** *In vitro* osteoclast survival after RANKL and M-CSF removal (n=3 mice per group). Scale bars, 200μm. **g,** Quantification of the osteoclast survival shown in **f**. *P* values were calculated using Student's *t*-test. Data are presented as the mean ± S.D. **h,** Principal coordinate analysis (PCoA) performed on osteoclasts derived from control or *Prmt5*$^{flox/\Delta}$ *Ctsk*-Cre mice. **i,** MA plot of the genes that were significantly differentially expressed in osteoclasts derived from control or *Prmt5*$^{flox/\Delta}$ *Ctsk*-Cre mice (light blue dots). **j,** The mRNA expression levels of *Prmt5* and osteoclast marker genes in control and *Prmt5*$^{flox/\Delta}$ *Ctsk*-Cre cells on Day 0 (before RANKL stimulation), Day 3 (3 days after RANKL stimulation) and Day 5 (5 days after RANKL stimulation) in the osteoclast culture system (Day 0: n=2 and 3, Day 3: n=3, Day 5: n=3 mice per group). *P* values were calculated using Student's *t*-test. Data are presented as the mean ± S.D. Source data are provided as a Source Data file.

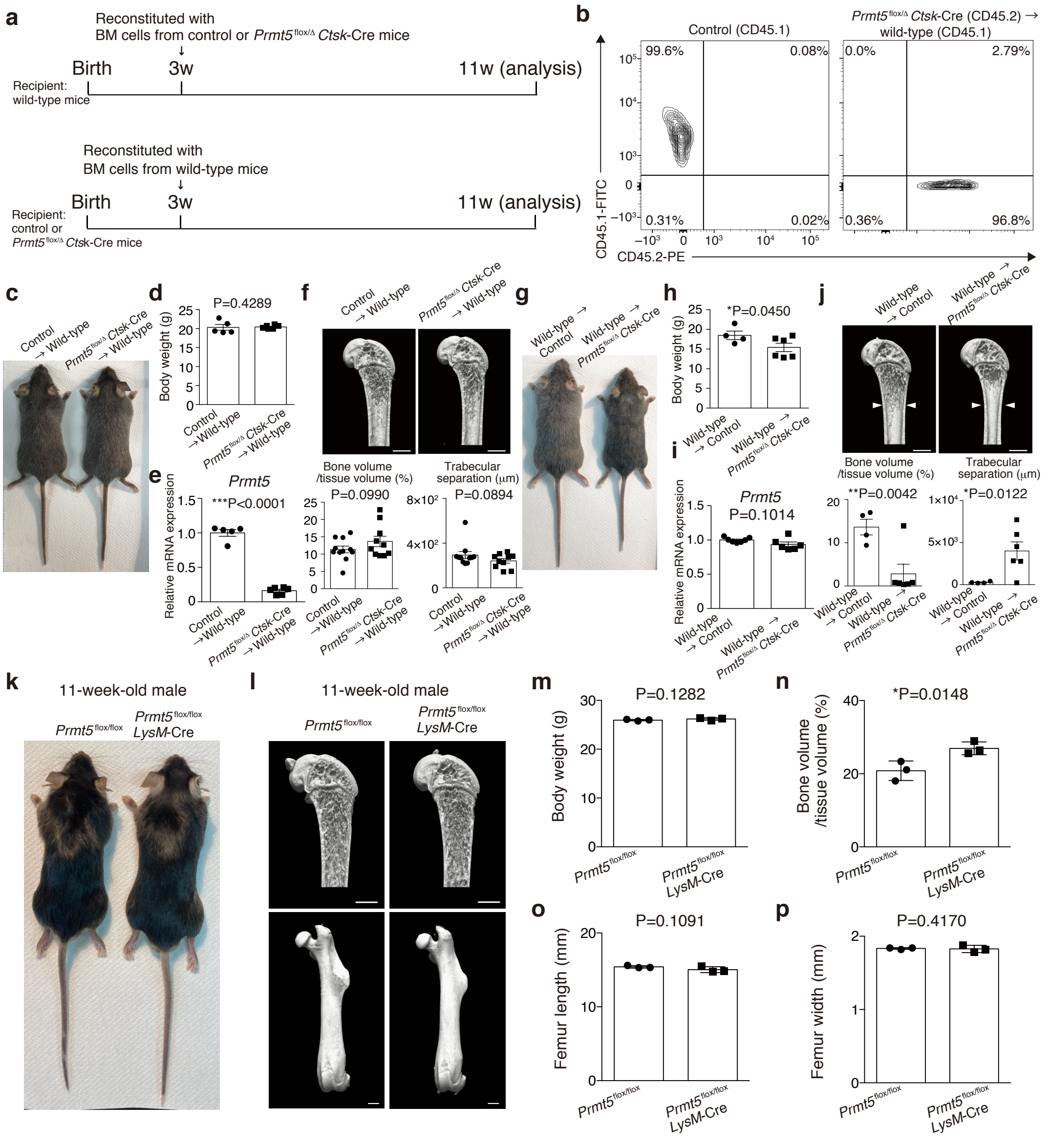

Supplementary Figure 4. Osteoclasts are not responsible for the *Prmt5* flox/Δ *Ctsk*-Cre mouse phenotypes. a, Experimental settings for **b-j**. **b,** Representative FACS plot of more than three independent experiments. **c,** Images of the bone marrow chimeric mice. **d,** Body weight of the chimeric mice (n=5 and 6 mice per group). *P* value was calculated using one-sided Student's *t*-test. Data are presented as the mean ± S.D. **e,** *Prmt5* expression levels in osteoclasts (n=5 and 6 mice per group). *P* value was calculated using one-sided Student's *t*-test. Data are presented as the mean ± S.D. **f,** Representative μCT images and parameters of the femur in the bone marrow chimeric mice (n=10 and 11 mice per group). Scale bars, 1mm. *P* values were calculated using one-sided Student's *t*-test. Data are presented as the mean ± S.D. **g,** Images of the bone marrow chimeric mice. **h,** Body weight of the chimeric mice (n=4 and 6 mice per group). *P* value was calculated using one-sided Student's *t*-test. Data are presented as the mean ± S.D. **i,** *Prmt5* expression levels in osteoclasts (n=6 and 7 mice per group) *P*

value was calculated using one-sided Student's *t*-test. Data are presented as the mean ± S.D. **j,** Representative μCT images and parameters of the femur in the bone marrow chimeric mice (n=4 and 6 mice per group). White arrow heads: bone width in the control mice. Scale bars, 1mm. *P* values were calculated using one-sided Student's *t*-test. Data are presented as the mean ± S.D. **k,** Images of 11-week-old male littermates. **l,** Representative femur images analyzed in **n-p**. Scale bars, 1mm. **m,** Body weight of 11-week-old male littermates (n=3 mice per group). *P* value was calculated using one-sided Student's *t*-test. Data are presented as the mean ± S.D. **n,** Bone volume/tissue volume in femur of the littermates (n=3 mice per group). *P* value was calculated using one-sided Student's *t*-test. Data are presented as the mean ± S.D. **o,** Femur length of the littermates (n=3 mice per group). *P* value was calculated using one-sided Student's *t*-test. Data are presented as the mean ± S.D. **p,** Femur width of the littermates (n=3 mice per group). *P* value was calculated using one-sided Student's *t*-test. Data are presented as the mean ± S.D. Source data are provided as a Source Data file.

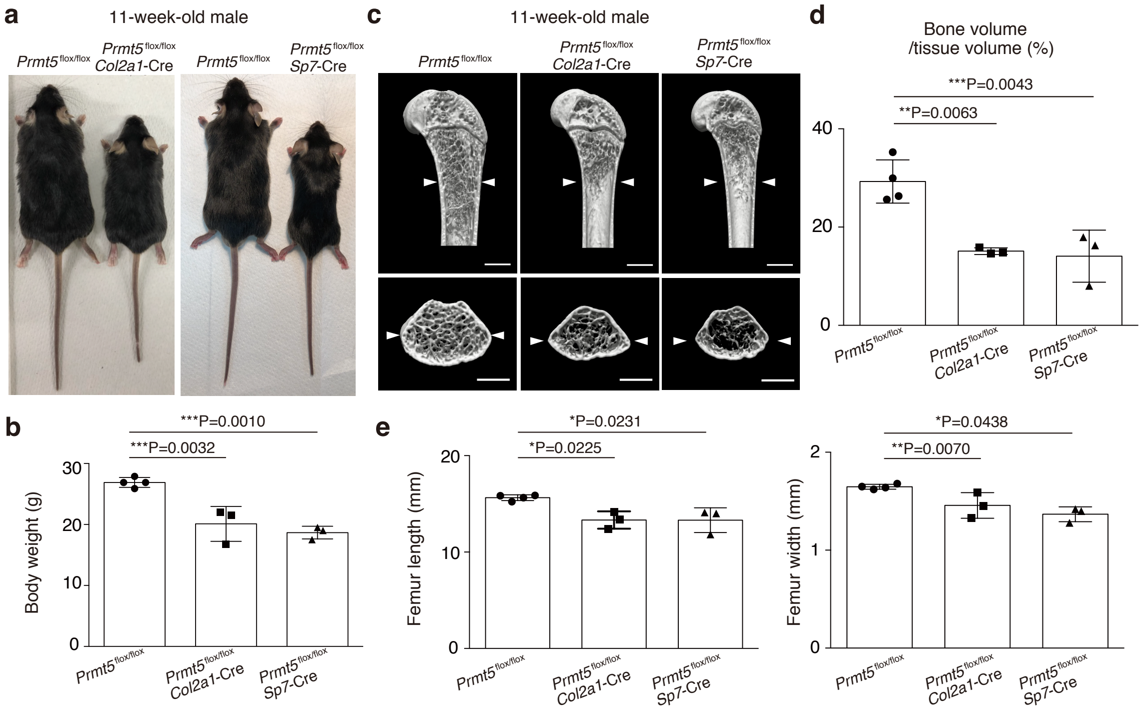

Supplementary Figure 5. Deletion of Prmt5 in skeletal progenitors recapitulates skeletal phenotypes of *Prmt5*$^{\mathbf{flox/\Delta}}$ *Ctsk*-Cre mice. a, Macroscopic image of 11-week-old male *Prmt5*$^{\text{flox/flox}}$, *Prmt5*$^{\text{flox/flox}}$ *Sp7*-Cre and *Prmt5*$^{\text{flox/flox}}$ *Col2a1*-Cre mice. **b,** Body weight of 11-week-old male *Prmt5*$^{\text{flox/flox}}$ (n=4 mice per group), *Prmt5*$^{\text{flox/flox}}$ *Sp7*-Cre (n=3 mice per group) and *Prmt5*$^{\text{flox/flox}}$ *Col2a1*-Cre (n=3 mice per group) mice. *P* values were calculated using ANOVA with Dunnett's multiple-comparison test. Data are presented as the mean ± S.D. **c,** Representative μCT images of the femur from 11-week-old male *Prmt5*$^{\text{flox/flox}}$, *Prmt5*$^{\text{flox/flox}}$ *Sp7*-Cre and *Prmt5*$^{\text{flox/flox}}$ *Col2a1*-Cre mice. The white arrow heads indicate bone width in the control mice. Scale bars, 1mm. **d,** Bone volume per tissue volume in femur of 11-week-old male *Prmt5*$^{\text{flox/flox}}$ (n=4 mice per group), *Prmt5*$^{\text{flox/flox}}$ *Sp7*-Cre (n=3 mice per group) and *Prmt5*$^{\text{flox/flox}}$ *Col2a1*-Cre (n=3 mice per group) mice. *P* values were calculated using ANOVA with Dunnett's multiple-comparison test. Data are presented as the mean ± S.D. **e,** Femur length and femur width of 11-week-old male *Prmt5*$^{\text{flox/flox}}$ (n=4 mice per group), *Prmt5*$^{\text{flox/flox}}$ *Sp7*-Cre (n=3 mice per group) and *Prmt5*$^{\text{flox/flox}}$ *Col2a1*-Cre (n=3 mice per group) mice. *P* values were calculated using ANOVA with Dunnett's multiple-comparison test. Data are presented as the mean ± S.D. Source data are provided as a Source Data file.

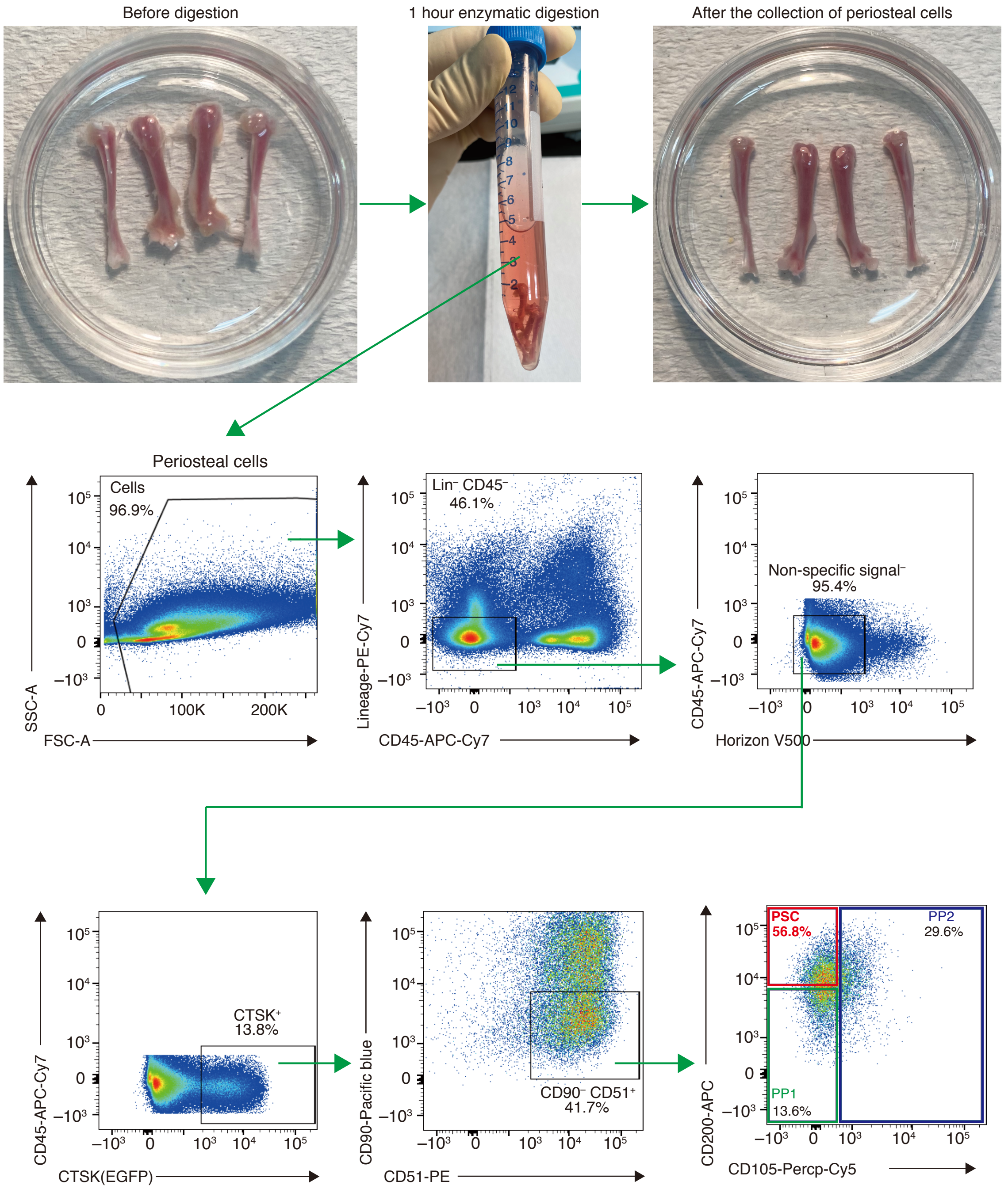

Supplementary Figure 6. The method for periosteal cell correction and FACS gating strategy for PSC analysis. Long bones without muscles (upper left panel) from *Prmt5*$^{flox/+}$ *Ctsk*-Cre CAG-CAT-EGFP mice were subjected to enzymatic digestion for 1 hour at 37 °C with agitation. After the removal of the long bones without periosteum (upper right panel), the tubes were centrifuged to harvest periosteal cells. The periosteal cells were subjected to FACS using the gating strategy shown in this figure.

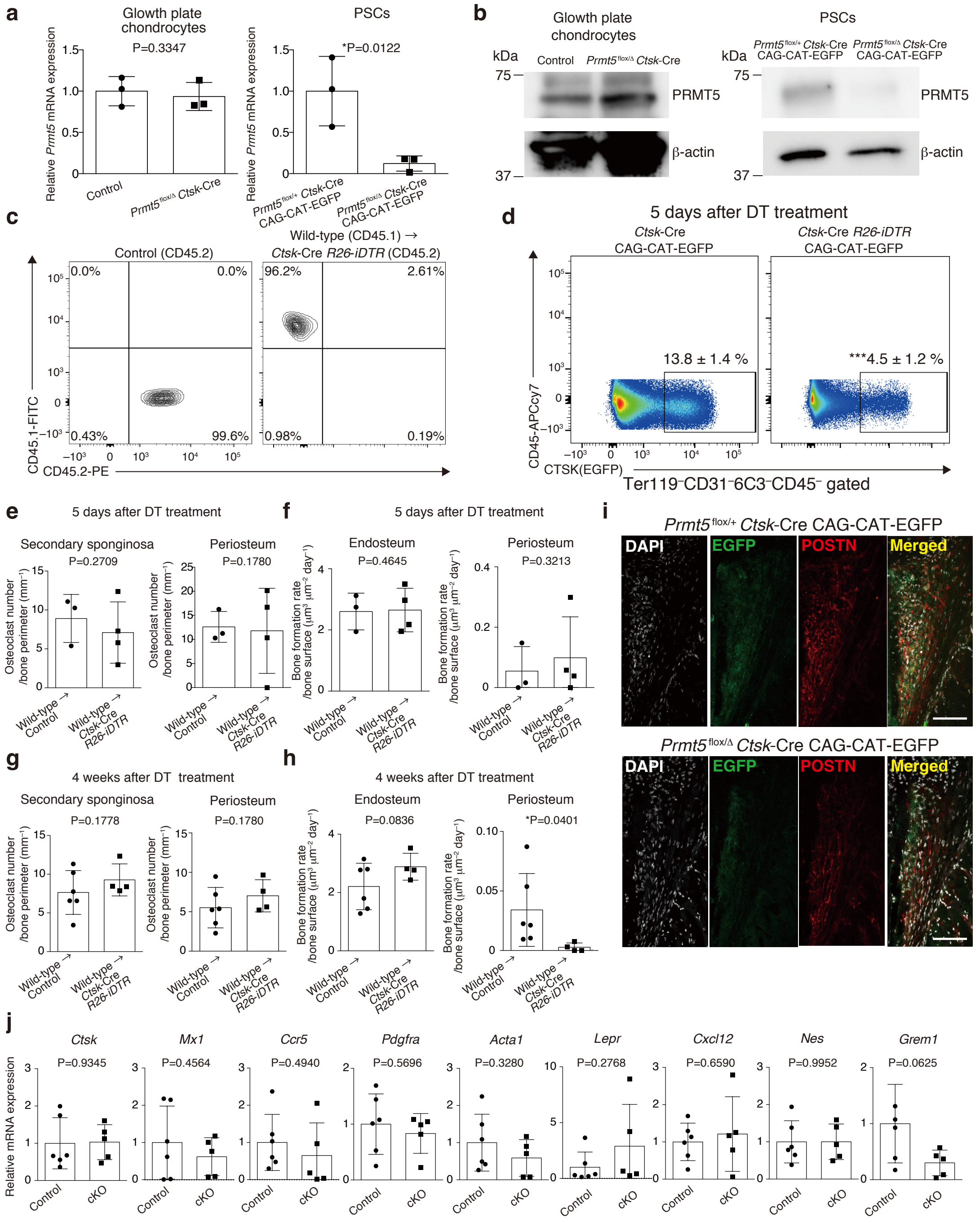

Supplementary Figure 7. The specific deletion of Prmt5 in PSCs and the consecutive effects of PSC deletion on bone metabolism. a, The expression levels of *Prmt5* mRNA in growth plate chondrocytes and PSCs (n=3 mice per group). *P* values were calculated using Student's *t*-test. Data are presented as the mean ± S.D. **b,** The expression levels of PRMT5 protein in growth plate chondrocytes and PSCs. Representative pictures of two independent experiments are shown. **c,** FACS plot showing the engraftment rates in the bone marrow transfer system. Representative data of more than three independent experiments are shown. **d,** FACS plot showing the

deletion efficiency of CTSK-expressing periosteal osteogenic progenitors 5 days after the diphtheria toxin (DT) treatment (n=3 mice per group). P=0.0005. *P* value was calculated using Student's *t*-test. **e,** Osteoclast number/bone perimeter in secondary sponginosa and periosteum 5 days after the bone marrow transfer and diphtheria toxin treatment (n=3 and 4 mice per group). *P* values were calculated using one-sided Student's *t*-test. Data are presented as the mean ± S.D. **f,** Bone formation rate per bone surface in the periosteum and endosteum 5 days after the bone marrow transfer and diphtheria toxin treatment (n=3 and 4 mice per group). *P* values were calculated using one-sided Student's *t*-test. Data are presented as the mean ± S.D. **g,** Osteoclast number/bone perimeter in secondary sponginosa and periosteum 4 weeks after the bone marrow transfer and diphtheria toxin treatment (n=6 and 4 mice per group). *P* values were calculated using one-sided Student's *t*-test. Data are presented as the mean ± S.D. **h,** Bone formation rate/bone surface in the periosteum and endosteum 4 weeks after the bone marrow transfer and diphtheria toxin treatment (n=6 and 4 mice per group). *P* values were calculated using one-sided Student's *t*-test. Data are presented as the mean ± S.D. **i,** Non-merged images of Fig3f. Scale bars, 100 μm. **j,** The mRNA expression levels of markers of periosteal osteogenic progenitors and skeletal stem cells in PSCs derived from Control (*Prmt5*$^{flox/+}$ *Ctsk*-Cre CAG-CAT-EGFP) (n=6 mice per group) and cKO (*Prmt5*$^{flox/\Delta}$ *Ctsk*-Cre CAG-CAT-EGFP) mice (n=5 mice per group). *P* values were calculated using two-sided Student's *t*-test. Data are presented as the mean ± S.D. Source data are provided as a Source Data file.

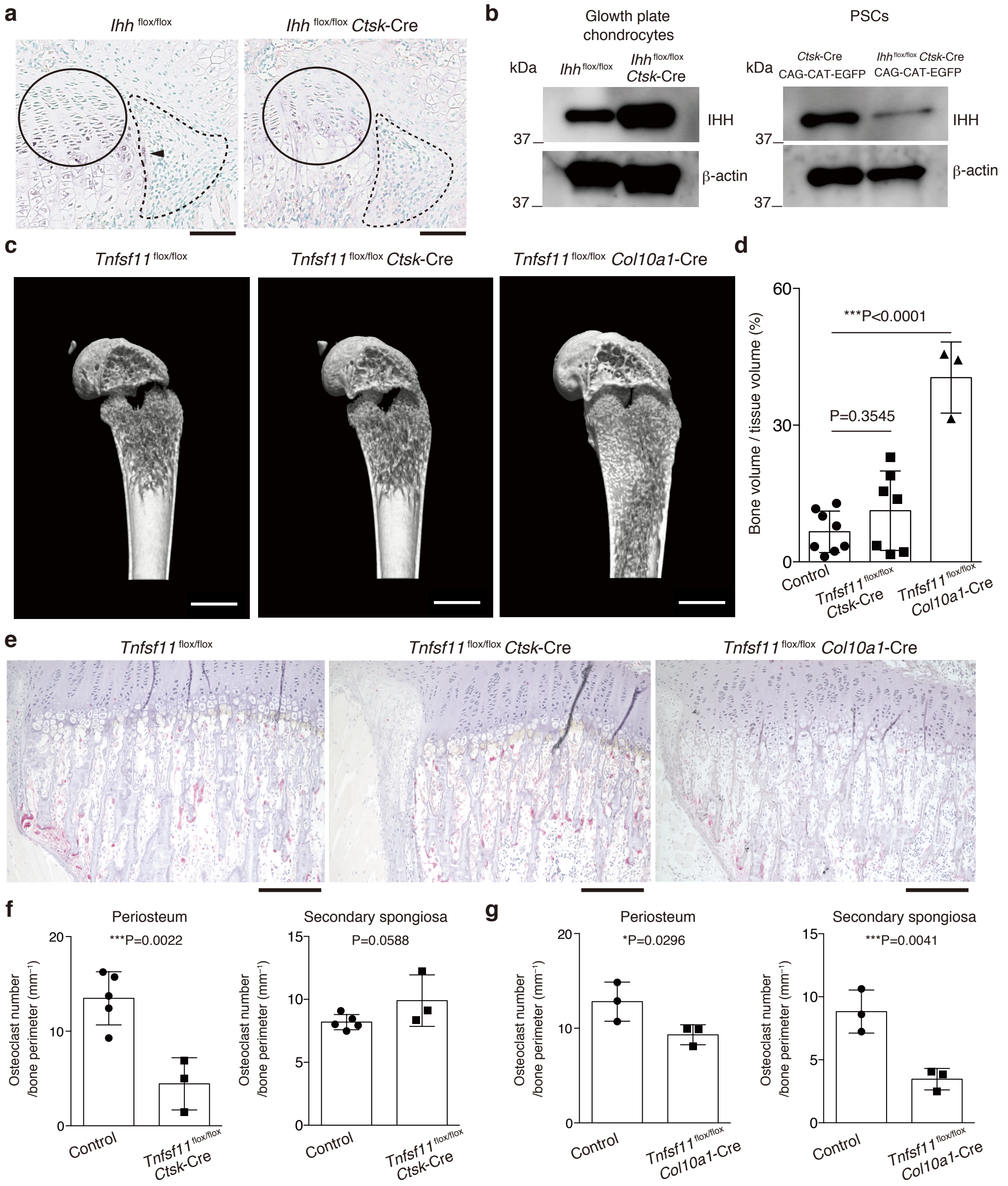

Supplementary Figure 8. Cre recombination does not occur in hypertrophic chondrocytes in the *Ctsk*-Cre system. a, *In situ* hybridization of *Ihh* mRNA in the growth plate and the adjacent periosteal region in 4-week-old male *Ihh*$^{flox/flox}$ and *Ihh*$^{flox/flox}$ *Ctsk*-Cre mice. Black dot lines indicate the periosteal region adjacent to the growth plate. The black arrowhead indicates *Ihh* mRNA expressing cell in the periosteal region. The *Ihh* mRNA expression decreased in the periosteal region, but not in the growth plate in *Ihh*$^{flox/flox}$ *Ctsk*-Cre mice. Scale bars, 100μm. Representative images of more than three independent experiments are shown. **b,** The expression levels of IHH protein in growth plate chondrocytes and PSCs derived from control and *Ihh*$^{flox/flox}$ *Ctsk*-Cre mice. Representative pictures of two independent experiments are shown. **c,**

Representative μCT images of the femur from 3-week-old female *Tnfsf11*$^{flox/flox}$, *Tnfsf11*$^{flox/flox}$ *Ctsk*-Cre and *Tnfsf11*$^{flox/flox}$ *Col10a1*-Cre mice. Representative pictures of more than three independent experiments are shown. Scale bars, 1mm. **d,** Bone volume per tissue volume in femur of 3-week-old female control (*Tnfsf11*$^{flox/flox}$ and *Tnfsf11*$^{flox/+}$, n=8 mice per group) *Tnfsf11*$^{flox/flox}$ *Ctsk*-Cre (n=7 mice per group) and *Tnfsf11*$^{flox/flox}$ *Col10a1*-Cre (n=3 mice per group) mice. *P* values were calculated using ANOVA with Dunnett's multiple-comparison test. Data are presented as the mean ± S.D. **e,** TRAP staining of the proximal tibiae of 3-week-old female *Tnfsf11*$^{flox/flox}$, *Tnfsf11*$^{flox/flox}$ *Ctsk*-Cre and *Tnfsf11*$^{flox/flox}$ *Col10a1*-Cre mice. Representative pictures of more than three independent experiments are shown. Scale bars, 200μm. **f,** The osteoclast number per bone perimeter in secondary sponginosa and periosteum measured by bone morphometric analysis in 3-week-old female control (n=5 mice per group) and *Tnfsf11*$^{flox/flox}$ *Ctsk*-Cre (n=3 mice per group) mice. *P* values were calculated using one-sided Student's *t*-test. Data are presented as the mean ± S.D. **g,** The osteoclast number per bone perimeter in secondary sponginosa and periosteum measured by bone morphometric analysis in 3-week-old male control and *Tnfsf11*$^{flox/flox}$ *Col10a1*-Cre mice (n=3 mice per group). *P* values were calculated using one-sided Student's *t*-test. Data are presented as the mean ± S.D. Source data are provided as a Source Data file.

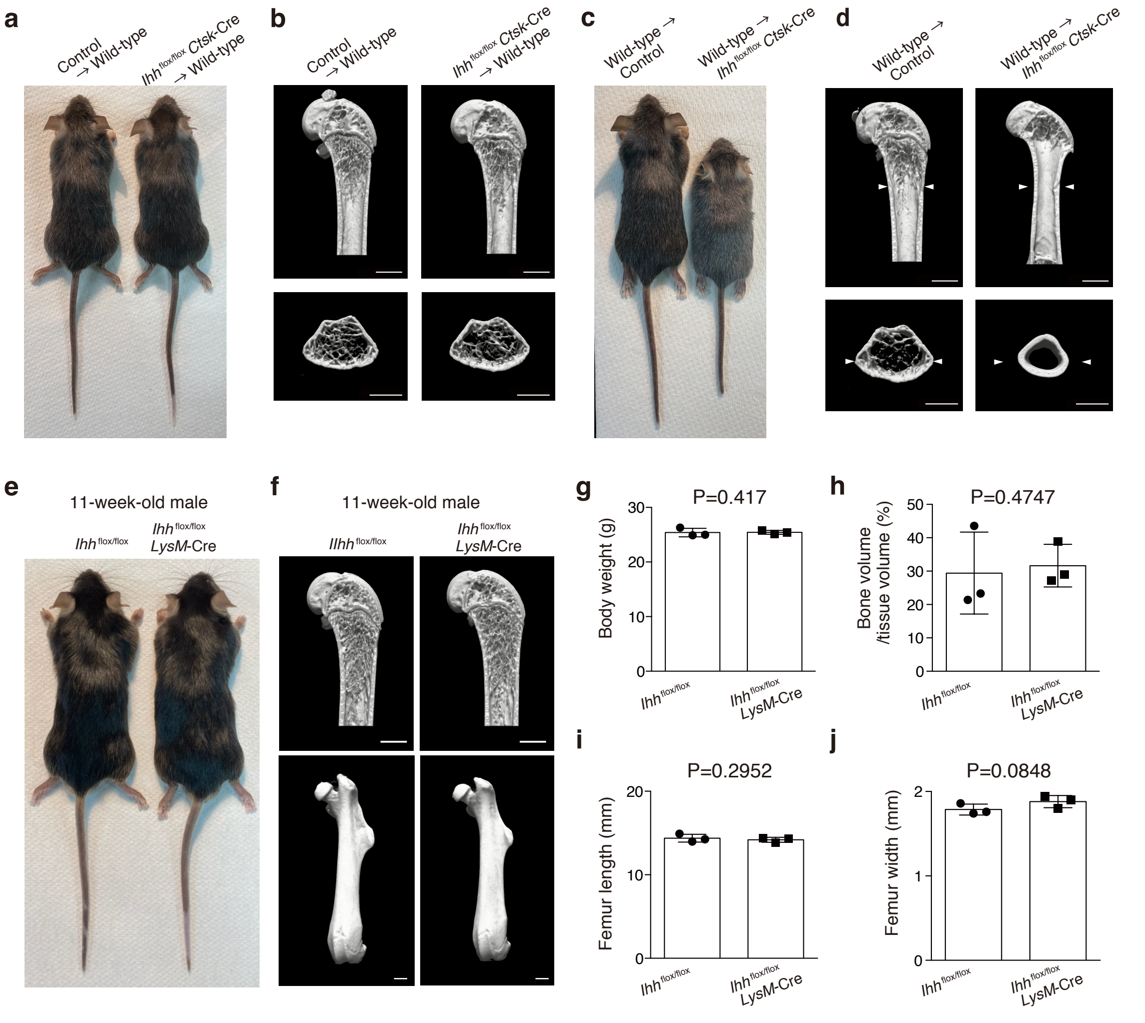

Supplementary Figure 9. Non- haematopoietic cells are responsible for the skeletal phenotype in *Ihh*$^{flox/flox}$ *Ctsk*-Cre mice. a, Macroscopic image of wild-type mice transferred with control (*Ihh*$^{flox/flox}$) or *Ihh*$^{flox/flox}$ *Ctsk*-Cre bone marrow cells. 3-week-old recipient mice were sub-lethally irradiated and administered an injection of bone marrow cells, and analyzed 8 weeks later as same as other bone marrow transfer experiments performed in this study. Representative data of more than two independent experiments is shown. **b,** Representative μCT images of the femur in the bone marrow chimeric mice shown in **a**. Representative data of more than two independent experiments is shown. Scale bars, 1mm. **c,** Macroscopic image of control (*Ihh*$^{flox/+}$ *Ctsk*-Cre) or *Ihh*$^{flox/flox}$ *Ctsk*-Cre mice transferred with wild-type bone marrow cells. Representative data of more than two independent experiments is shown. **d,** Representative μCT images of the femur in control (*Ihh*$^{flox/+}$ *Ctsk*-Cre) or *Ihh*$^{flox/flox}$ *Ctsk*-Cre mice transferred with wild-type bone marrow cells. The white arrow heads indicate bone width in the control mice. Representative data of more than two independent experiments is shown. Scale bars, 1mm. **e,** Macroscopic image of 11-week-old male *Ihh*$^{flox/flox}$ and *Ihh*$^{flox/flox}$ *LysM*-Cre mice. **f,** Representative μCT

images of the femur from 11-week-old male *Ihh*$^{flox/flox}$ and *Ihh*$^{flox/flox}$ *LysM*-Cre mice. Representative pictures of more than three independent experiments are shown. Scale bars, 1mm. **g,** Body weight of 11-week-old male *Ihh*$^{flox/flox}$ and *Ihh*$^{flox/flox}$ *LysM*-Cre mice (n=3 mice per group). *P* value was calculated using one-sided Student's *t*-test. Data are presented as the mean ± S.D. **h,** Bone volume per tissue volume in femur of 11-week-old male *Ihh*$^{flox/flox}$ and *Ihh*$^{flox/flox}$ *LysM*-Cre mice (n=3 mice per group). *P* value was calculated using one-sided Student's *t*-test. Data are presented as the mean ± S.D. **i,** Femur length of 11-week-old male *Ihh*$^{flox/flox}$ and *Ihh*$^{flox/flox}$ *LysM*-Cre mice (n=3 mice per group). *P* value was calculated using one-sided Student's *t*-test. Data are presented as the mean ± S.D. **j,** Femur width of 11-week-old male *Ihh*$^{flox/flox}$ and *Ihh*$^{flox/flox}$ *LysM*-Cre mice (n=3 mice per group). *P* value was calculated using one-sided Student's *t*-test. Data are presented as the mean ± S.D. Source data are provided as a Source Data file.

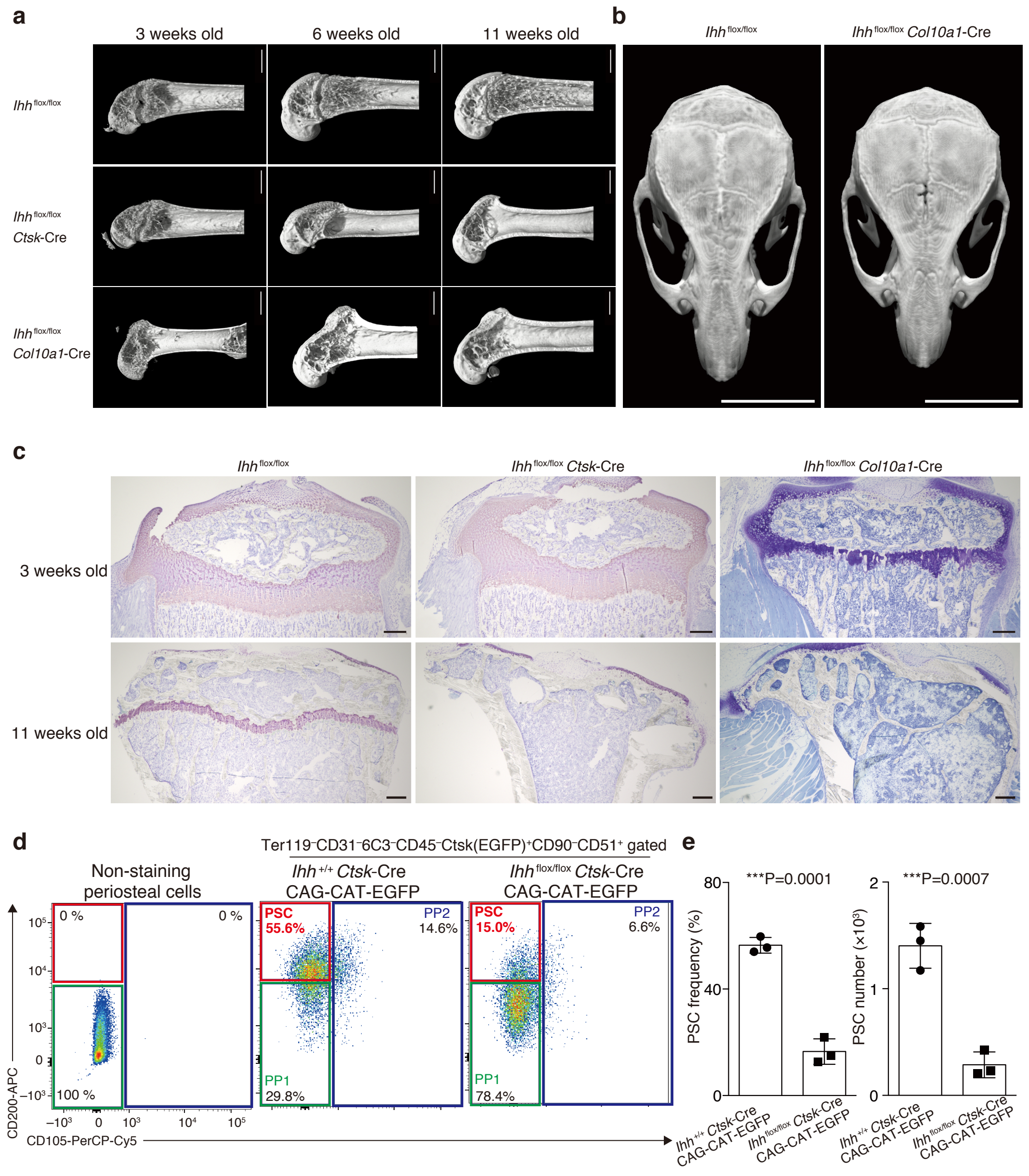

Supplementary Figure 10. A comparative analysis of skeletal phenotypes in *Ihh*$^{flox/flox}$ *Ctsk*-Cre mice and *Ihh*$^{flox/flox}$ *Col10a1*-Cre mice. a, Representative µCT images of the femur in female *Ihh*$^{flox/flox}$, *Ihh*$^{flox/flox}$ *Ctsk*-Cre and *Ihh*$^{flox/flox}$ *Col10a1*-Cre mice at 3, 6 and 11 weeks of age. Representative data of more than two independent experiments is shown. Scale bars, 1mm. **b,** µCT analysis of the skull of female littermates at the age of 11 weeks. Representative pictures of more than three independent experiments are shown. Scale bars, 7mm. **c,** Toluidine blue staining of the proximal tibiae of female *Ihh*$^{flox/flox}$, *Ihh*$^{flox/flox}$ *Ctsk*-Cre and *Ihh*$^{flox/flox}$ *Col10a1*-Cre mice at 3 and 11 weeks of age. Representative data of more than two independent experiments is shown. Scale bars, 200 µm. **d,** Frequency of periosteal osteogenic

progenitors (PSC, PP1 and PP2) in the long bones of *Ihh*$^{+/+}$ *Ctsk*-Cre CAG-CAT-EGFP and *Ihh*$^{flox/flox}$ *Ctsk*-Cre CAG-CAT-EGFP mice. Non-staining periosteal cells were used to set the gating. Representative data of a triplicate experiment are shown. **e,** The frequency and number of PSC in the long bones of *Ihh*$^{+/+}$ *Ctsk*-Cre CAG-CAT-EGFP and *Ihh*$^{flox/flox}$ *Ctsk*-Cre CAG-CAT-EGFP mice (n=3 mice per group). *P* values were calculated using one-sided Student's *t*-test. Data are presented as the mean ± S.D. Source data are provided as a Source Data file.

PRMT5

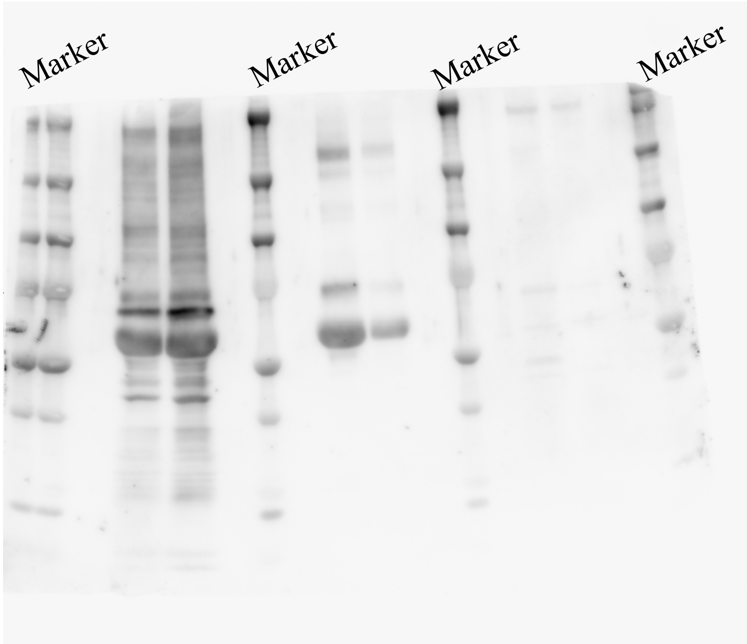

β-actin

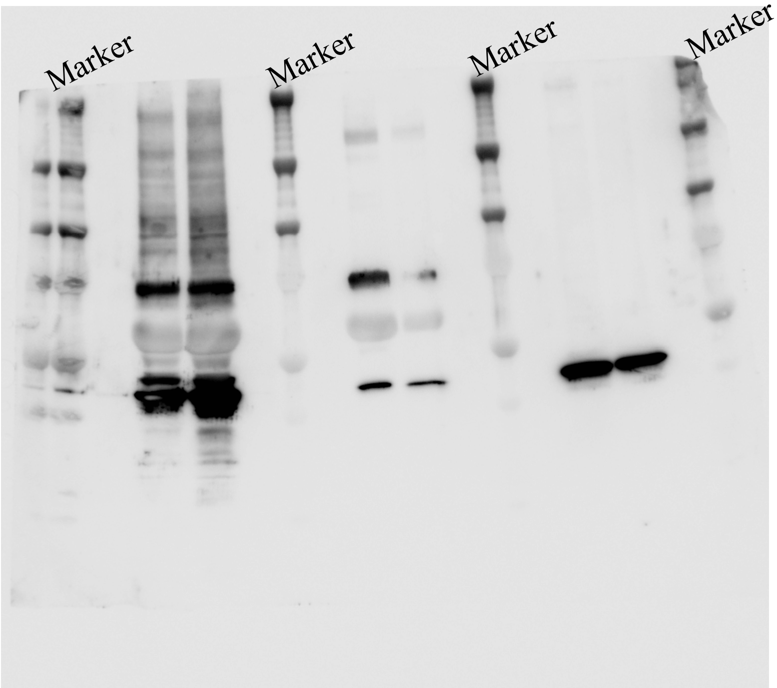

Supplementary Figure 11. Uncropped blots for supplementary figure 7b.

IHH

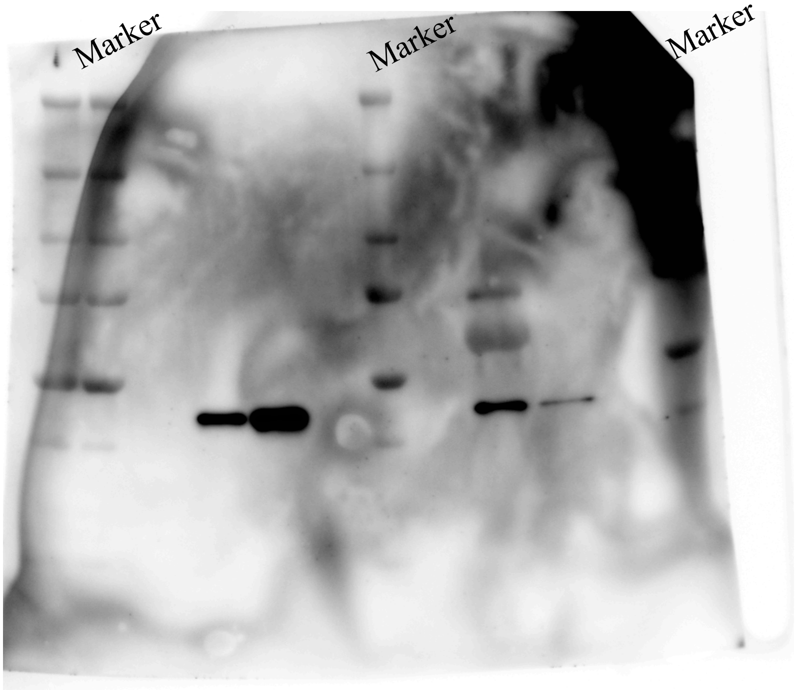

β-actin

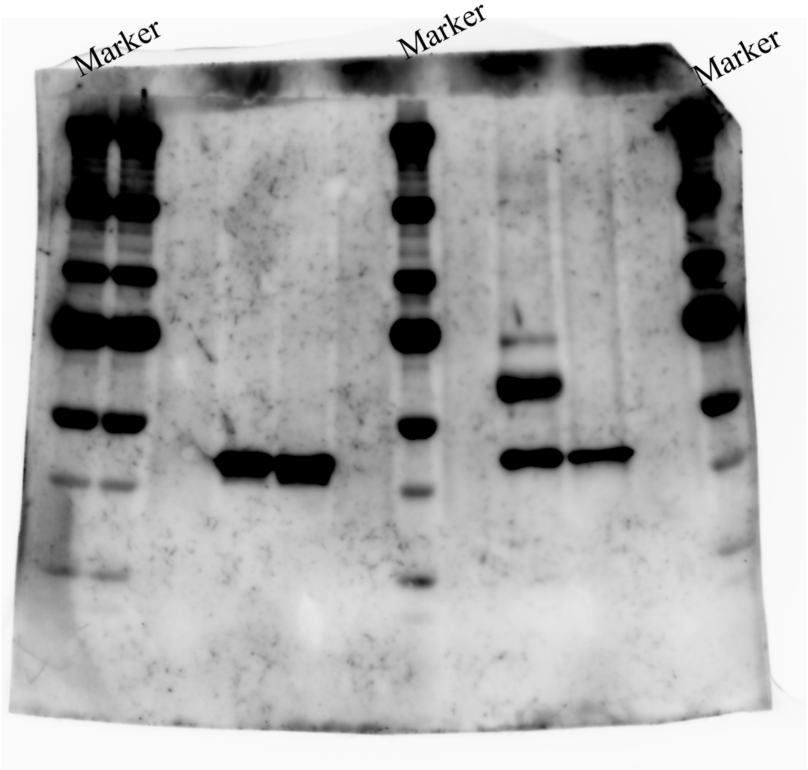

Supplementary Figure 12. Uncropped blots for supplementary figure 8b.
