# Supplementary material for: Periosteal stem cells control growth plate stem cells during postnatal skeletal growth
Source: Nat Commun. 2022 Jul 18;13:4166. doi: 10.1038/s41467-022-31592-x (PMC9293991; doi:10.1038/s41467-022-31592-x)
Supplement: Supplementary file 2 — Reporting Summary [file 41467_2022_31592_MOESM2_ESM.pdf]

nature research

Corresponding author(s): Hiroshi Takayanagi

Last updated by author(s): May 30, 2022

Reporting Summary

Nature Research wishes to improve the reproducibility of the work that we publish. This form provides structure for consistency and transparency in reporting. For further information on Nature Research policies, see Authors & Referees and the Editorial Policy Checklist .

Statistics

For all statistical analyses, confirm that the following items are present in the figure legend, table legend, main text, or Methods section.

n/a	Confirmed	
☐	☒	The exact sample size (*n*) for each experimental group/condition, given as a discrete number and unit of measurement
☐	☒	A statement on whether measurements were taken from distinct samples or whether the same sample was measured repeatedly
☐	☒	The statistical test(s) used AND whether they are one- or two-sided<br>*Only common tests should be described solely by name; describe more complex techniques in the Methods section.*
☒	☐	A description of all covariates tested
☐	☒	A description of any assumptions or corrections, such as tests of normality and adjustment for multiple comparisons
☐	☒	A full description of the statistical parameters including central tendency (e.g. means) or other basic estimates (e.g. regression coefficient) AND variation (e.g. standard deviation) or associated estimates of uncertainty (e.g. confidence intervals)
☐	☒	For null hypothesis testing, the test statistic (e.g. *F*, *t*, *r*) with confidence intervals, effect sizes, degrees of freedom and *P* value noted<br>*Give P values as exact values whenever suitable.*
☒	☐	For Bayesian analysis, information on the choice of priors and Markov chain Monte Carlo settings
☒	☐	For hierarchical and complex designs, identification of the appropriate level for tests and full reporting of outcomes
☒	☐	Estimates of effect sizes (e.g. Cohen's *d*, Pearson's *r*), indicating how they were calculated

Our web collection on statistics for biologists contains articles on many of the points above.

Software and code

Policy information about availability of computer code

Data collection	Diva software v9.0.1 (BD Biosciences), BZ-II Analyzer BZ-H2A v.1.42 (Keyence), Multi Gauge software ver.3.0 (FUJIFILM).
Data analysis	FlowJo V9.9.3 software (TreeStar) , Graph Pad Prism V5 and V7 (GraphPad Software), FastQC (v0.1.1.8), TrimGalore (v0.6.4), Kallisto (v0.46.0), DESeq2 (v1.26)

For manuscripts utilizing custom algorithms or software that are central to the research but not yet described in published literature, software must be made available to editors/reviewers. We strongly encourage code deposition in a community repository (e.g. GitHub). See the Nature Research guidelines for submitting code & software for further information.

Data

Policy information about availability of data

All manuscripts must include a data availability statement. This statement should provide the following information, where applicable:

- Accession codes, unique identifiers, or web links for publicly available datasets
- A list of figures that have associated raw data
- A description of any restrictions on data availability

The RNAseq data produced in this study were deposited to the public data base (GSE146872: https://www.ncbi.nlm.nih.gov/geo/query/acc.cgi?acc=GSE146872). We also used the bulk-RNA seq data for periosteal progenitors (GSE106235:https://www.ncbi.nlm.nih.gov/geo/query/acc.cgi?acc=GSE106235) and the mouse transcriptome index (mus musculus GRCm38.96: https://www.ncbi.nlm.nih.gov/assembly/GCF_000001635.20/). Source data are provided with this paper.

Field-specific reporting

Please select the one below that is the best fit for your research. If you are not sure, read the appropriate sections before making your selection.

- [x] Life sciences
- [ ] Behavioural & social sciences
- [ ] Ecological, evolutionary & environmental sciences

For a reference copy of the document with all sections, see nature.com/documents/nr-reporting-summary-flat.pdf

Life sciences study design

All studies must disclose on these points even when the disclosure is negative.

Sample size	No sample-size calculation was performed. The sample sizes were guided by previous studies using similar bone analyses (Asano et al., Nature Metabolism 1, 868–875, 2019, Hayashi et al., Nature, 485(7396), 69-74, 2012).
Data exclusions	No data were excluded from the analyses.
Replication	Experiments were repeated at least once unless otherwise noted. The number of biological replicates (n) is reported for each experiment. All attempts at replication were successful.
Randomization	The order of bone analyses for different genotypes was randomized. Beyond this, assigning animals to experimental groups is not relevant to this study, as the groups are defined by genotype and no disease models were used. Animals of different sexes were analyzed independently to remove covariate.
Blinding	Blinding was used for bone histomorphometric and micro-CT analyses. Blinding to group allocation during data collection is not relevant to this study because he groups are defined by genotype and no disease models were used.

Reporting for specific materials, systems and methods

We require information from authors about some types of materials, experimental systems and methods used in many studies. Here, indicate whether each material, system or method listed is relevant to your study. If you are not sure if a list item applies to your research, read the appropriate section before selecting a response.

Materials & experimental systems

n/a	Involved in the study	
☐	☒	Antibodies
☒	☐	Eukaryotic cell lines
☒	☐	Palaeontology
☐	☒	Animals and other organisms
☒	☐	Human research participants
☒	☐	Clinical data

Methods

n/a	Involved in the study	
☒	☐	ChIP-seq
☐	☒	Flow cytometry
☒	☐	MRI-based neuroimaging

Antibodies

Antibodies used

- PE anti-mouse CD51 (RMV-7, BioLegend, Cat# 104106, 1:100 dilution for FCM)
- APC anti-mouse CD200 (OX-90, Biolegend, Cat# 123810, 1:100 dilution for FCM)
- PB anti-mouse CD90.2 (53-2.1, Biolegend, Cat# 140306, 1:100 dilution for FCM)
- PerCP-Cy5.5 anti-mouse CD105 (MJ7/18, Biolegend, Cat# 120416, 1:50 dilution for FCM)
- APCcy7 anti-mouse CD45 (30-F11, Biolegend, Cat# 103116, 1:100 dilution for FCM)
- Biotinylated anti-mouse Ter119 (TER-119, Biolegend, Cat# 116204, 1:100 dilution for FCM)
- Biotinylated anti-mouse CD31 (390, Biolegend, Cat# 102404, 1:100 dilution for FCM)
- Biotinylated anti-mouse Ly-51 (6C3, Biolegend, Cat# 108304, 1:100 dilution for FCM)
- PEcy7 streptavidin (B278254, Biolegend, Cat# 405206, 1:100 dilution for FCM)
- FITC anti-mouse CD73 (TY/11.8, Biolegend, Cat# 127220, 1:100 dilution for FCM)
- PE anti-mouse CD49e (MFR5, Biolegend, Cat# 103805, 1:100 dilution for FCM)
- APC anti-mouse Ki67 (16A8, Biolegend, Cat# 652406, 1:100 dilution for FCM)
- PE anti-mouse Flk2 (A2F10, Biolegend, Cat# 135306, 1:100 dilution for FCM)
- APC anti-mouse Sca-1 (D7, Biolegend, Cat# 108112, 1:100 dilution for FCM)
- eFluor 450 anti-mouse CD34 (RAM34, eBioscience, Cat# 48-0341-82, 1:100 dilution for FCM)
- PerCP-Cy5.5 anti-mouse CD150 (mShad150, eBioscience, Cat# 46-1502-82, 1:100 dilution for FCM)
- PE-Cy7 anti-mouse CD48 (HM48-1, Biolegend, Cat# 103424, 1:100 dilution for FCM)

- APCcy7 anti-mouse CD117/c-kit (2B8, Biolegend, Cat# 105826, 1:100 dilution for FCM)
- FITC streptavidin (eBioscience, Cat# 11-4317-87, 1:100 dilution for FCM)
- PE anti-mouse CD16/32 (93, Biolegend, Cat# 101308, 1:100 dilution for FCM)
- PE anti-mouse Ter119 (TER-119, Biolegend, Cat# 116208, 1:100 dilution for FCM)
- FITC anti-mouse CD71 (RI7217, Biolegend, Cat# 113806, 1:100 dilution for FCM)
- Biotinylated anti-mouse CD3ε (145-2C11, Biolegend, Cat# 100304, 1:100 dilution for FCM)
- Biotinylated anti-mouse CD4 (RM4-5, Biolegend, Cat# 100508, 1:100 dilution for FCM)
- Biotinylated anti-mouse CD8a (53-6.7, Biolegend, Cat# 100704, 1:100 dilution for FCM)
- Biotinylated anti-mouse CD11b (M1/70, Biolegend, Cat# 101204, 1:100 dilution for FCM)
- Biotinylated anti-mouse CD11c (HL3, BD, Cat# 553800, 1:100 dilution for FCM):
- Biotinylated anti-mouse CD45R/B220 (RA3-6B2, Biolegend, Cat# 103204, 1:100 dilution for FCM)
- Biotinylated anti-mouse Gr-1 (RB6-8C5, Biolegend, Cat# 108404, 1:100 dilution for FCM)
- Biotinylated anti-mouse CD49b (DX5, Biolegend, Cat# 108904, 1:100 dilution for FCM)
- FITC anti-mouse CD45.1 (A20, Biolegend, Cat# 110706, 1:100 dilution for FCM)
- PE anti-mouse CD45.2 (104, Biolegend, Cat# 109808, 1:100 dilution for FCM)
- Anti-mouse periostin (Abcam, Cat# ab14041, 1:100 dilution for immunohistochemistry)
- Anti-GFP (Invitrogen, Cat# A10262, 1:100 dilution for immunohistochemistry)
- Anti-β-actin (Sigma-Aldrich, Cat# A5441, 1:1000 dilution for western blotting)
- Anti-PRMT5 (Santa Cruz , PRMT5-21, Cat# sc-59650, 1:200 dilution for western blotting)
- Anti-IHH (Abcam, Cat# ab52919, 1:200 dilution for western blotting)

Validation

All antibodies used here are commercially available. Antibodies for FACS purchased from BioLegend are quality control tested by immunofluorescent staining with flow cytometric analysis as mentioned in manufacturer's website. Antibodies for FCM purchased from eBioscience were verified by Cell treatment to ensure that the antibody binds to the antigen stated as mentioned in manufacturer's website. Antibody for FACS purchased from BD is validated by routinely testing for flow cytometric analysis as mentioned in manufacturer's website. Antibodies for immunohistochemistry purchased from Abcam and Invitrogen are quality control tested for immunohistochemistry as mentioned in manufacturer's website. Antibodies for western blotting purchased from Sigma-Aldrich, Santa Cruz and Abcam are quality control tested for western blotting as mentioned in manufacturer's website

Animals and other organisms

Policy information about studies involving animals; ARRIVE guidelines recommended for reporting animal research

Laboratory animals

All animals were maintained under specific pathogen-free conditions, and all experiments were performed with the approval of the Institutional Review Board at The University of Tokyo. C57BL/6 mice were purchased from CLEA Japan. KSN/Slc Nude mice (6-8 week-old male) were purchased from SLC Japan. R26-iDTR mice and Ihh flox/flox mice were obtained from the Jackson Laboratory. Prmt5 flox/flox mice (Inoue et al., Nature Immunology 2018), Ctsk-Cre mice(Nakamura et al., Cell 2007) and CAG-CAT-EGFP mice(Kawamoto et al., FEBS Letter 2000) were described previously. The sex and age of mice used are described in figures or figure legends. All animals were maintained at a constant ambient temperature of 22-26 degree Celsius, 40-65% of humidity under a 12 h light/dark cycle with free access to food and drink.

Wild animals

The study did not involve wild animals.

Field-collected samples

The study did not involve samples collected from the field.

Ethics oversight

All experiments were performed with the approval of the Animal Ethics Committee of The University of Tokyo.

Note that full information on the approval of the study protocol must also be provided in the manuscript.

Flow Cytometry

Plots

Confirm that:

- [x] The axis labels state the marker and fluorochrome used (e.g. CD4-FITC).
- [x] The axis scales are clearly visible. Include numbers along axes only for bottom left plot of group (a 'group' is an analysis of identical markers).
- [x] All plots are contour plots with outliers or pseudocolor plots.
- [x] A numerical value for number of cells or percentage (with statistics) is provided.

Methodology

Sample preparation	Isolation of periosteal progenitor cells was adapted and modified from a previously described protocol (Debnath et al., Nature 2018). Long bones including periosteum were collected and then digested without mincing in alpha-MEM with collagenase (1mg/ml), Dispase2 (2mg/ml) and DNAse1 (1mg/ml) at 37°C for 1h. Cell suspensions were passed through a 100µm cell strainer, washed with HBSS containing 10% FBS and stained with antibodies against Ter119, CD31, 6C3, CD51, CD90.2, CD45, CD105 and CD200.
Instrument	FACS Aria (BD Bioscience)
Software	FACS DIVA software (BD Biosciences) and FlowJo V10.0.7 software (TreeStar)
Cell population abundance	Post-sort purity was not determined
Gating strategy	Periosteal stem cells (CD45-Ter119-CD31-6C3-CD51+CD90.2-CD105-CD200+) were sorted by using previously-defined gating strategies (Chan, C. K., et al., Cell, 2015, Debnath et al., Nature 2018).

☒ Tick this box to confirm that a figure exemplifying the gating strategy is provided in the Supplementary Information.
